# If you don’t let it in, you don’t have to get it out: Thought preemption as a method to control unwanted thoughts

**DOI:** 10.1371/journal.pcbi.1010285

**Published:** 2022-07-14

**Authors:** Isaac Fradkin, Eran Eldar

**Affiliations:** 1 Department of Psychology, Hebrew University of Jerusalem, Jerusalem, Israel; 2 Department of Cognitive and Brain Sciences, Hebrew University of Jerusalem, Jerusalem, Israel; Peking University, CHINA

## Abstract

To attain goals, people must proactively prevent interferences and react to interferences once they occur. Whereas most research focuses on how people deal with external interferences, here we investigate the use of proactive and reactive control in dealing with unwanted thoughts. To examine this question, we asked people to generate an association to each of several repeating cue words, while forbidding the repetition of associations. Reactively rejecting and replacing unwanted repeated associations after they occur entails slower response times. Conversely, proactive control entails constricting the search space and thus faster response times. To gain further insight into different potential proactive thought control mechanisms, we augmented the analysis of raw response times with a novel, hypothesis-based, tractable computational model describing how people serially sample associations. Our results indicate that people primarily react to unwanted thoughts after they occur. Yet, we found evidence for two latent proactive control mechanisms: one that allows people to mitigate the episodic strengthening of repeated thoughts, and another that helps avoid looping in a repetitive thought. Exploratory analysis showed a relationship between model parameters and self-reported individual differences in the control over unwanted thoughts in daily life. The findings indicate the novel task and model can advance our understanding of how people can and cannot control their thoughts and memories, and benefit future research on the mechanisms responsible for unwanted thought in different psychiatric conditions. Finally, we discuss implications concerning the involvement of associative thinking and various control processes in semantic fluency, decision-making and creativity.

## Author summary

Trying to stop thinking unwanted, often repetitive thoughts is a familiar experience. However, being aware of such attempts implies that the thought has already reached consciousness. Can we preempt an unwanted thought from coming to mind, similarly to how we can avoid taking an unwanted action? We examined this question using a free association task where people were instructed to avoid repeating associations. We show that to meet the task, people principally reject and replace unwanted associations after they have already reached consciousness. This rejection, however, is effective at ensuring that the same unwanted associations do not continue to come to mind endlessly. Furthermore, we found that people are able to control the extent to which associations become temporarily stronger when they repeat. Individual differences in these abilities could explain the difficulties that some individuals experience with unwanted and intrusive thoughts.

"An ounce of prevention is worth a pound of cure."

Benjamin Franklin

## Introduction

Often, a particular cue can repeatedly evoke unwanted thoughts or memories. For example, a particular song or object can remind us of a painful, past romantic relationship, which we may not want to think of. People report using different strategies to control such unwanted thoughts or memories (e.g., trying to suppress the thought) [1–3]. Such conscious attempts for thought control reflect, by definition, a reactive process, occurring after an unwanted thought has already disturbed us (henceforth: *reactive thought control*). The current study investigates our ability for *proactive thought control*. Returning to the example above, can one preempt break-up-related unwanted thoughts or memories from coming to mind when encountering cues associated with the lost relationship? Furthermore, even when trying to reactively distract oneself from painful memories evoked, for instance, by a particular song, what mechanism can ensure that the same unwanted memory will not continue to come up over and over again? More generally, studying proactive thought control mechanisms has important implications for understanding how we avoid constant interference by unrelated thoughts, and why some people experience more intrusive, repetitive, or simply task-irrelevant thoughts than others [4–8].

Whereas proactive control has been investigated for decades [9,10], the recent *dual mechanisms of control framework* [11,12] offers a comprehensive account of the distinction between proactive and reactive cognitive control. Proactive control includes sustained anticipatory or very early selection mechanisms, biasing attention, and perception towards current goals. Conversely, reactive control corresponds with a transient, ’late correction’ mechanism, kicking in only after the unwanted event. These mechanisms are assumed to involve partially dissociated neural mechanisms [11,13] and different behavioral signatures. Specifically, whereas reactively inhibiting interfering stimuli slows down responses, proactive control can help avoid increasing response times (RTs) [13,14]. Proactive control thus guarantees smoother performance with less interruptions, but it is resource-demanding, and depends on the predictability of possible interferences [14,15].

We are not aware of any investigations directly contrasting proactive and reactive *thought* control. Interestingly, however, this distinction is implied by the two main accounts of the classical white bear phenomenon, wherein asking people to suppress a specific thought often has the paradoxical effect of increasing its frequency [16,17]. Reactive thought control is implicated in this phenomenon by the suggestion that people comply with the request to suppress the thought by trying to distract themselves when the thought comes to mind [18]. However, it has also been suggested that the to-be-suppressed thought is monitored by a continuous, unconscious process [19], potentially preempting the thought from reaching consciousness altogether. It thus remains unclear whether thought control in this task, or in any other setting, is primarily reactive or proactive, or involves an interaction between the two. Indeed, empirically investigating the generation and control of naturally occurring, daily thought is a challenging endeavor.

To simplify the problem, we focus here on thought control in the specific case of associative memory retrieval. Tasks probing associative memory are a useful tool for investigating thought processes, as evidenced by the relationship between people’s responses in a free association task and their everyday thoughts [20]. Numerous studies have demonstrated people’s ability to intentionally forget memories learned in the lab. A classic example is offered by *directed forgetting* experiments, showing that people are able to voluntarily forget specific words or entire lists of words upon instruction [21,22]. Efficient forgetting is also observed in the *think-no-think* paradigm. In this paradigm, participants are trained to produce a specific word upon presentation of a specific cue [23]. Then, participants are shown each cue and are instructed to either think or suppress thoughts of its associated word. The typical finding is reduced memory of suppressed words on a later recall test. Although it is still debated whether this reduced memory reflects interference, weakening of associative binding, or weakening of the memory itself [24–27], these findings imply that people can intentionally weaken the strength of associations formed in the lab. Critically, evidence for intentional forgetting of long-term (e.g., autobiographical) memories has been either absent [28] or limited to paradigms wherein such memories were linked to unrelated cues in the lab [29–31]. Thus, whether people can prevent predominant, long-term memories from coming to mind is unclear. Extant intentional forgetting paradigms are inherently unsuitable for answering this question because they measure recall accuracy and long-term memories are unlikely to be completely forgotten even after intensive suppression attempts.

The idea that semantic cognition involves a variety of specialized control mechanisms has been extensively studied with respect to the processing of externally presented stimuli [32–36]. To date, most such studies have focused on reactive control mechanisms. For example, delayed responses to a word (e.g., *fork*) that is weakly related to a prime preceding it (e.g., *table*) engage reactive control mechanisms required for inhibiting the dominant meaning of the prime (e.g., *table*-*chair*) [37]. However, a recent study has shown that when people know in advance what aspects of a semantic stimulus they ought to focus on (e.g., the use of an object vs. its physical features) they can proactively gate predominant associations from interfering with the processing of task-relevant stimuli [38]. Although this preliminary finding resonates with the idea that proactive control depends on the extent to which the stimuli to-be-ignored are predictable [12,14,39], these and other studies of semantic control examined the processing of externally-presented stimuli rather than the generation of thoughts or associations.

Thus, despite the great interest in control mechanisms in episodic and semantic memory, whether people can preempt specific consolidated associations from coming to mind remains unknown. For this purpose, we introduce a novel paradigm examining the frequency and speed with which people *spontaneously* recall unwanted associations. In this modified free association task, participants are presented with verbal cues, each repeating several times, and are instructed to not repeat associations they already had in response to previous presentations of a cue (*suppress group*). To achieve this goal, participants may either proactively preempt the repeated associations from coming to mind in the first place, or reactively seek an alternative association each time a repeated association comes to mind. The degree to which participants use either method can be determined by comparing the produced associations and the speed with which they are produced to those of a *control group* to whom repeated associations are not forbidden. By focusing on association frequency and speed, as opposed to the binary measure of accuracy employed in instructed recall paradigms, this task offers a much more sensitive test of proactive control over long-term memories.

**Fig 1:**
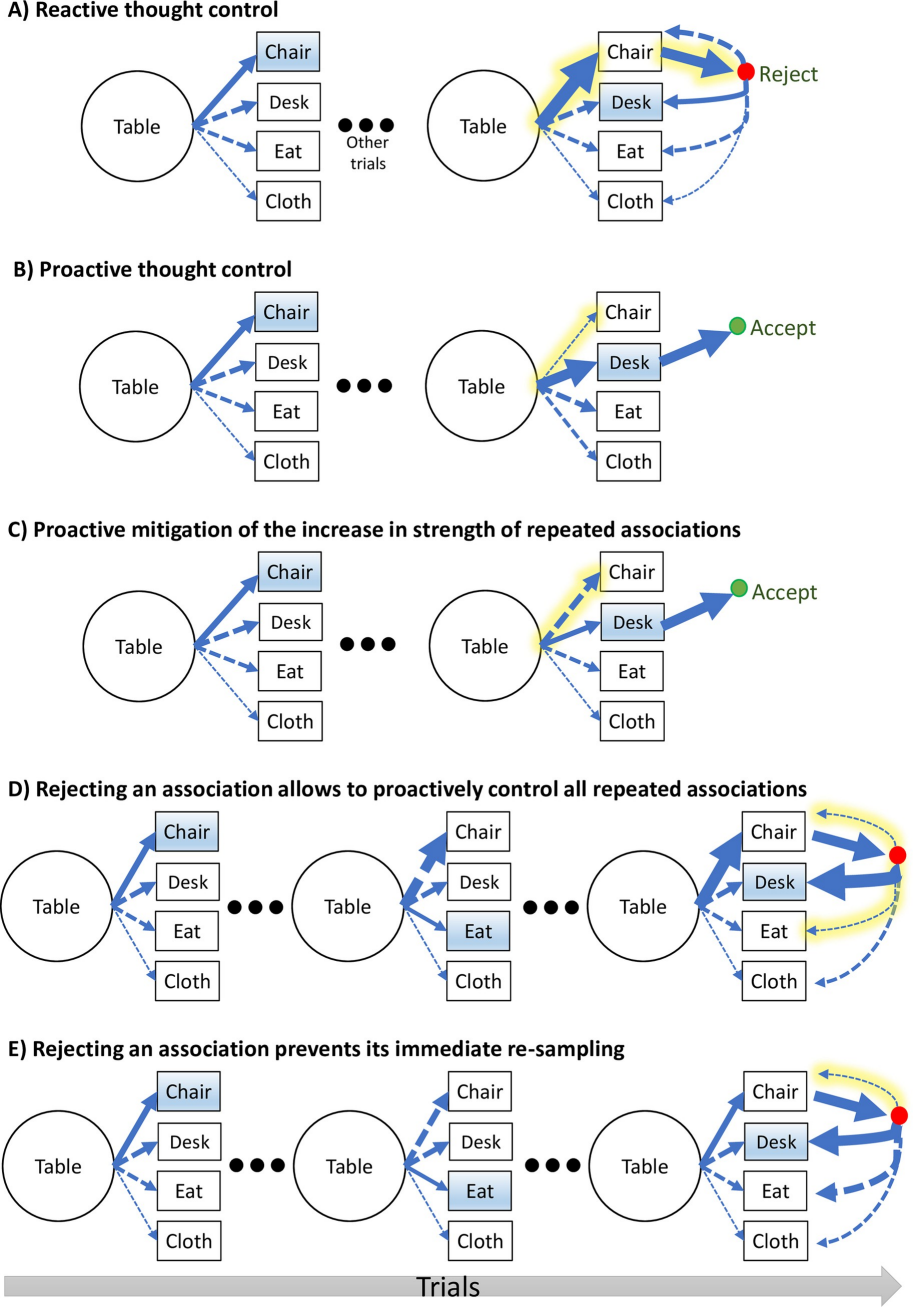
Possible mechanisms for avoiding associations generated in previous trials. The figure illustrates pure reactive (A), and pure proactive thought control (B), as well three mechanisms of latent proactive control (C-E). Each row displays in the rightmost trial how one (`Chair`in panels A-C) or two (`Chair`and `Eat`in panels D-E) prior associations are avoided in favor of a new association (‘Desk`). Partial latent proactive control may be achieved by mitigating the natural, episodic enhancement of repeated associations (compare panel C with panel A), or by triggering proactive control after a first candidate association is rejected (panels D and E). The latter, post-rejection mechanism may be able to prevent any repeated association (D) or only the association that has just been rejected (E). Line thickness corresponds to associative strength. Dashed lines represent possible associations whereas solid lines represent the actual enacted policy. Reported associations are highlighted in blue. The unique characteristics of each solution are highlighted in yellow, shown only for the rightmost trials, together with green circles denoting acceptance and reporting of an association and red circles denoting rejection of a generated association, leading to an attempt to think of an alternative association.

Reactive thought control entails one central prediction–that the need to reject a repeated association (`Chair`in Fig 1A) and come up with a new association (`Desk`) delays the response. Thus, new associations to repeated cues (i.e., *new/repeated trials*) will be slower than associations to cues presented for the first time (i.e., *new/new trials*). One caveat here is that new/repeated trials tend to involve weaker associations (since the associations that have already been reported tend to be strong), and weaker associations are expected to be slower regardless of whether repeated associations are forbidden [40]. Thus, a more specific prediction entailed by reactive thought control is of a more pronounced slowdown in the suppress group compared to the control group.

Conversely, proactive thought control in our task may be implemented by means of several different mechanisms. One possible mechanism involves the reduction of the associative strength of an association once it is recalled for the first time (`Chair`in Fig 1B). This entails a facilitation of new associations to repeated cues (new/repeated RT < new/new RT; compare the thickness of the arrow pointing at `Desk`in the rightmost trial to that of the leftmost trial in Fig 1B), because preempting repeated associations implies a restriction of the search space, and thus less competition [40–42]. However, even if people are unable to weaken associations’ associative strength, they might be able to enact more subtle proactive control by mitigating a potential temporary strengthening of an association due to the fact that it just recently came to mind (compare the thickness of the arrow pointing at `Chair`in the rightmost trial in Fig 1C to the one in Fig 1A). The plausibility of this latter mechanism is supported by the above discussed findings of intentional forgetting of recently formed links.

Another, latent, proactive mechanism may rely on the predictability of specific interferences, which seems a key condition for proactive control over the processing of external stimuli [11,12,14,38]. Whereas it is impossible to keep in mind all of the associations previously generated for *any* cue [43], focusing on associations previously given to a specific, predictable cue could allow for the proactive gating of these associations. Say, for example, that a participant reported the associations `Chair`and `Eat`to previous presentations of the cue `Table`, and after several additional cues were presented, the cue ‘Table’ is presented again. The random order of the cues makes each presentation of ‘Table’ unpredictable, and this could make it difficult to proactively avoid the associations `Chair`and `Desk`. However, once the participant has reactively rejected one repeated association (e.g., `Chair`), they are now faced with the task of generating an alternative association to the same, this time predictable, cue. Consequently, the participant may now be able to proactively inhibit repeated associations (`Chair`and `Eat`; see Fig 1D), or at least those associations that have already been rejected in the current trial (`Chair`). A yet more limited possibility is that a participant is at least able to proactively avoid re-sampling an association immediately after its rejection, in a manner that may resemble classical predictability-based sensory gating mechanisms [44,45] (Fig 1E). This latter process could be relevant for understanding the difficulty that some people have in stopping a particular thought, and thus enter an endless, ruminative loop [46].

Examining raw RTs alone is insufficient for determining to what degree participants used each of the above mechanisms. Indeed, even if proactive control could be made possible after an initial rejection of an association (Fig 1D and 1E), this initial rejection is still expected to prolong RTs, even though preventing the need for further rejections will lead to a lesser delay than what might be predicted under pure reactive control. Similarly, even if participants might be able to fully avoid the natural increase in the associative strength of previously reported associations (Fig 1C), if these associations are naturally strong, they will still be likely to emerge in repeated presentation of a cue and require time-consuming reactive control. Thus, to investigate these latent modes of proactive control we developed a novel computational framework formalizing the process by which an association is sampled and then either accepted or rejected in favor of sampling an alternative association.

Several previous models have formulized memory recall as a sequential, Markovian process in which some (e.g., repeated) memories remain unreported (i.e., rejected) [47–49]. These models have been primarily applied to tasks asking participants to generate multiple responses to a cue (e.g., fluency task). Here we extend this approach to model the latent control processes preceding the report of a single association. Furthermore, whereas previous models have overlooked the fact that some associations are stronger than others, and that stronger associations take less time to recall, here we use a Semi-Markov process (SMP) model that allows states (i.e., associations) to vary in the time it takes to generate them, while retaining tractability. This allows distinguishing two plausible causes for increases in RT to repeated cues: repeated associations coming to mind and being rejected, and weaker association being generated because the strongest and fastest ones have already been generated to previous presentation of the cue. Dissociating these processes is necessary for our main goal: examining the degree to which avoiding repeated associations involves a) changes in the associative strength of repeated associations, b) rejection of repeated associations after they are generated, and c) changes in the associative strength of repeated associations *immediately after* the rejection of the first repeated association.

Importantly, whereas here we apply the SMP to the specific case of thought control in free association, this model may be useful for elucidating the mechanisms involved in any task in which proactive and reactive control interact, as well as other tasks involving sequential memory retrieval. For example, decision-makers faced with a realistic open-ended decision problem (e.g., how to spend a weekend) first need to generate their options, a process that has been formulized as a random-walk over semantic memory [50]. Furthermore, the interaction between semantic retrieval and control has been implicated in creativity research [51,52], yet a formal model is missing. We discuss these and other implications in the discussion.

## Method

### Ethics statement

The study was approved by the ethics committee of the faculty of Social Sciences of the Hebrew University of Jerusalem (Approval number 130120), and all participants provided written informed consent, as required by the ethics approval.

### Participants

Ninety-seven participants were recruited via the Prolific Academic internet platform, using the following inclusion criteria: Adults (age ≥ 18), raised monolingual with English as first language, who reported having no language-related disorders, Dyslexia or ADHD, currently residing in an English-speaking country, and with a minimum approval rate of 97% from at least 200 previous studies and at most 10,000 submissions. Participants received monetary compensation for their participation (£7.5 for an experiment taking approximately 60 minutes) and an additional bonus for performing the task in accordance with the instructions (see below). 13 participants (13%) showing clear evidence of inattentive or negligible performance were excluded from further analyses based on preregistered criteria that are detailed below, in line with inattentive performance rates found in previous studies [53,54]. Data from four additional participants were removed due to a lack of variance in associative strength ratings which precluded further analysis (see S1 Text). A pre-registration of the study’s hypotheses, analysis plan, and sample size justifications, as well as the data and scripts used for analysis and modeling can be found at https://osf.io/nwy4f/.

The final sample included 40 participants in each group, as required by a preregistered power analysis based on expected effect sizes estimated in a small pilot study (N = 18). The pilot results showed a larger new/repeated–new/new RT difference for the suppress group (Cohen’s *d* = 1.58). To account for the possibility that this effect was overestimated, we simulated the percentage of times power would reach or exceed the intended level if the power analysis was to be reproduced many times (i.e., assurance; [55]). Using an assurance level of 95%, our sample size ensured a power of 85% for detecting group differences in the new/repeated–new/new RT difference. Whereas the pilot data primarily supported the reactive thought control hypothesis, we also wanted to ensure that we have sufficient power to find support for the proactive thought control hypothesis. For this purpose, we fitted the SMP model (see `Computational Model`section below) to the control group pilot data and then manipulated either the parameter controlling proactive control, or the parameter controlling reactive control, to simulate data with rates of repeated associations similar to those found in the suppress group pilot data. This procedure indicated that the observed increase in repeated associations can be produced either by a standardized effect size of -2.04 in the main proactive control parameter or an effect size of 3.27 in the main reactive control parameter, suggesting that our sample size ensures a power of 99% for finding group differences in either parameter.

Participants included 39 women (48.75%) and 39 men (48.75%; 2 participants did not report gender) and were 39.65 *(SD* = 12.38) years old on average. The highest level of education acquired was high school for 30 participants (37.50%), a bachelor’s degree for 38 participants (47.50%), and a master’s degree or higher for seven participants (8.75%; 5 did not report education level). There were no significant group differences in age (*t* (77.78) = 0.47, *p* = 0.64), gender (χ^2^(2) = 3.89, *p* = 0.14), or education (χ^2^(3) = 1.62, *p* = 0.65).

## Materials and procedure

### Free association task

The free association task was administered over the internet using Gorilla Experiment Builder, which affords good precision in recording the timing of responses [56]. Participants were instructed to "write an association that comes to mind in response to the presented word." The task included 60 cues, each presented five times, in random order (see S2 Text for details on how cues were chosen). Participants in the suppress group were told that they would not get a monetary bonus for trials in which they repeat an association. Thus, for this group, repeated associations were followed by a red X in addition to a large, red ’thumbs-down’ icon to stress the importance of avoiding repeated associations. In addition, to ensure that participants in the suppress group did not attempt to avoid repetitions by writing non-words, grammatical variations on previous associations, or completely random words, both groups were requested to provide only highly related, common associations. In practice, a response matching any common association, as determined in previous norms [57], was considered correct. Thus, associations that did not appear in previous norms were followed by a red X.

Each trial consisted of a fixation cross presented for 1000±150ms, followed by a cue word (e.g., `Table`) presented at the center of the screen for 500ms. A key challenge in measuring RTs in typed responses is the significant contamination related to typing speed variability across participants and across keys. Thus, to dissociate the time it takes to think of an association from the time it takes to write it, we asked participants to first press the spacebar as soon as an association came to mind and only then type in their association and press enter. Participants were nudged to avoid premature presses by providing them with a relatively long time to press the spacebar (maximum 15s, indicated by a stopwatch on the screen) but very limited time to start typing once they pressed the spacebar (1300ms). If the participant did not start typing in time, the trial ended, and a message "Please press the spacebar only after you have a response" appeared on the screen in red.

To estimate the associative strength of reported associations, following the task, we asked participants to review their associations and rate on a visual analog scale, for each trial, "To what extent does the word [cue] reminds you of the word [the association]", with 0 corresponding to "not at all" and 1 to "very much". The two words were presented sequentially with a 500ms lag, so as to emphasize the directional nature of associative strength [58].

Several measures were taken to monitor and minimize negligent performance. First, the experiment was presented on the participants’ entire screen, and participants were not allowed to navigate away from it. Second, we inspected participants’ responses for careless responding (e.g., writing gibberish). Third, explicit attention checks were added to the rating phase, where participants were asked to respond in a specific manner when seeing the word ’attention’ as a cue. In addition, the rating phase included 30 new catch trials that were not taken from the participant’s associations but rather represented common examples of related (where the second word was the most common association, across participants, to the cue) and unrelated (where the second word never appeared as an association) pairs of words. Fourth, associative strength ratings were expected to correlate positively with the proportion of participants reporting the respective associations in previous norms [57]. Participants failing two or more of these attention checks (N = 11), or failing to respond in more than 30% of trials (N = 2), or with an exceptionally low variance in ratings (>90% of ratings were above 97; N = 4), were excluded. The latter exclusion criterion was not preregistered since this behavior was unexpected, and thus, we conducted a sensitivity analysis including these participants where possible (S1 Fig). Finally, we excluded a small number of trials (16 overall) with reaction time faster than 100ms.

### Thought Suppression Inventory–revised (TSI-R)

The Effective Suppression subscale of the TSI-R [59] measures the extent to which participants experience success in suppressing their unwanted thoughts (e.g., "I am able to suppress unpleasant thoughts"). We chose this scale because it effectively dissociates experienced success in thought control from other related constructs, such as the experienced frequency of unwanted thoughts [1], the need to engage in thought control, or the question of how people control their thoughts [3]. The TSI-R effective suppression (effSup) subscale shows satisfactory reliability (*r* = 0.73–0.79), and validity in predicting psychiatric symptoms involving difficulties in thought control [59].

### Computational modeling

The SMP model formulizes a process in which an individual sequentially generates candidate associations until one of them is accepted and reported. Following previous models of memory retrieval [47,48,60,61], we assume that the probability of generating an association *A*_*i*_ is proportional to its associative strength (*AS*_*i*_). After a participant reports an association to a given cue for the first time, its associative strength in subsequent trials might decrease (indicating proactive control designed to avoid generating this association in subsequent trials) or increase (due to rehearsal effects). These changes in associative strength are formalized by the *associative strength modification* parameter (*p*):

$$A{S\prime }_{i}=\{\begin{array}{l} e^{\rho }AS_{i}\;if\;A_{i}\;is\;repeated\\ \;AS_{i}\;if\;A_{i}\;is\;new \end{array}$$ (1)

Thus, if *ρ* > 0 repeated associations are strengthened, whereas *ρ* < 0 entails they are weakened.

The probability that each candidate association is sampled is given by normalizing *AS*′_*i*_:

$$p(A_{i})=\frac{AS_{i}^{\prime }}{\sum AS_{i}^{\prime }}$$ (2)

After an association is sampled, a participant decides whether to accept and report it or, if it is a repeated association, reject it and generate another association instead. A thought rejection parameter, α, controls the probability that repeated associations are rejected:

$$p(reject\;A_{i})=\{\begin{array}{l} \alpha \;if\;A_{i}\;is\;repeated\\ \;0\;if\;A_{i}\;is\;new \end{array}$$ (3)

where *α* is a free parameter [0,1], with higher values entailing a higher probability of rejecting repeating associations.

The overall RT per reported association is the sum of the time it takes to generate all candidate associations until an association is accepted, plus a non-decision time. Based on previous findings [40], we assume that stronger associations could be faster to generate than weaker ones. Thus, the time it takes to generate each candidate association (whether it is accepted or rejected) is drawn from a Gamma distribution, the average of which (*μ*_*i*_) is a function of the surprisal associated with sampling the association (−log[*p*(*A*_*i*_)]), such that weaker associations tend to be slower:

$$\mu_{i}=e^{S^{\left(\mu \right)}}∙{\left(-\log\left[{p(A}_{i}\right)]\right)}^{E^{\left(\mu \right)}}$$ (4)

This functional form assumes a mean RT of 0 in the hypothetical case of a cue with a single association (where *p*(*A*_1_) = 1), whereas *S*^*(μ)*^ and *E*^*(μ)*^ control the slope and curvature of the function linking normalized associative strength to mean RT. Other functional forms (e.g., strictly linear, fixing *E*^*(μ)*^ to 1) were tested and produced poorer parameter recovery and/or absolute fit (see S3 Text).

Two additional parameters are needed to complete the specification of response times: a parameter determining the minimum RT, and a parameter specifying the standard deviation of the Gamma distribution. The former is given by **τ** and corresponds with ‘non-decision time’, typically used in evidence accumulation models to account for the time it takes to perceive presented stimuli (i.e., the cue) and execute a response [62]. Given that the mean and standard deviation of response times are typically correlated [63], and to improve parameter recovery, the latter was specified using the following equation:

$$\sigma_{i}=\lambda ∙\mu_{i}$$ (5)

Where *λ* is a free parameter determining the relationship between the mean and standard deviation of the Gamma distribution (alternative parameterizations produced worse parameter recovery; S3 Text). Together, these equations are used to specify two matrices: (a) a matrix defining the probabilities of transitioning between states (e.g., from the cue, or a rejected association, to the next candidate association), and (b) a matrix of parameters determining how long it takes to complete each transition. These matrices are used to derive the joint probability density function of the empirical RT and the reported association, using a Laplace transformation ([64]; note that the use of a Gamma distribution to model sampling times was motivated accordingly by its relatively simple Laplace transformation). Additional details regarding the specification and fitting of the SMP, including parameter and model recovery tests, can be found in S3 Text.

The remaining challenge in using these models for free association is mapping the space of possible associations for each cue. This requires estimating the number of the possible associations a participant can sample from and the strength of each association. Previous models have relied on the distribution of associations across participants for this purpose [48,65]. However, although participants were asked to give only common associations, almost half of the associations did not appear in previous norms (Median: 48.22%), highlighting that association spaces sampled across participants do not approximate within-participant association spaces very well. Thus, although we assume that any normed association could have been reported by each participant in our study, we also estimate the number of associations not appearing in previous norms (henceforth: *non-normed associations*). This estimate is tailored to each cue and participant based on the empirical proportions of non-normed associations reported for that cue (by all participants) and by that participant (for all cues). Then, the strengths of reported associations are given by participants’ associative strength ratings. To estimate associative strength for unreported normed associations, we assume it can be derived from their frequency in the population. Finally, we estimate the strength of unreported non-normed associations based on the distribution of normed associations and the empirical proportions of non-normed associations reported for each level of associative strength. Additional details regarding this method, as well as evidence for its validity are provided in S4 Text.

We conclude this section by specifying how the different modes of control depicted in Fig 1 manifest in the SMP. Reactive control (Fig 1A) is entailed in the model by a high rejection parameter (*α* >>0), whereas proactive control (Fig 1B) is entailed by a reduction in the associative strength of repeated associations (*ρ* < 0). If participants in the suppress group do not enact proactive control yet are able to mitigate the increase in strength of repeated associations due to rehearsal (Fig 1C), *ρ* values in this group will be non-negative but lower than *ρ* value in the control group, with *ρ* = 0 corresponding to full mitigation. To determine whether proactive control is employed only after an initial reactive rejection (Fig 1D), we tested a variant of the model with two associative strength modification parameters: one affecting repeated associations in the beginning of a trial (*ρ*) and one affecting them after an initial rejection (*ρ*_w_). Finally, we determined whether participants were able to avoid re-sampling an association that had just been rejected (Fig 1E) using a variant of the model that implements this constraint (a model where immediate re-sampling was controlled by a continuous parameter was not identifiable). Note that this implies rejected associations cannot be re-sampled only on the subsequent step. Ideally, we would also have tested a model where rejected associations can never be sampled again on the same trial, but since we used a Markovian process, this was not feasible.

## Results

Before examining the main predictions, we conducted a manipulation check examining whether the groups differed in the number of repeated associations. As expected, participants in the suppress group generated substantially fewer repeated associations (inter-quartile range, IQR = [6.05%, 14.85%]) than participants in the control group (IQR = [50.47%, 66.37%], *t* (51.76) = -16.22, *p* < .001, Cohen’s *d* = -3.63).

### Response time markers of reactive and proactive thought control

To examine whether the decrease in repeated associations to repeated cues in the suppress group was accompanied by faster or slower response times, we compared the two groups in terms of the difference in log RT when producing new associations to repeated, versus to new, cues. To reiterate, reactive thought control predicts that the process of rejecting a repeated association and generating a new one will result in longer RTs in responses to repeated cues (i.e., new/repeated trials), in comparison to the first presentation of a cue where no such rejection is needed (i.e., new/new trials; Fig 1A). Conversely, if participants proactively preempt repeated associations from coming to mind, new/repeated trials are expected to be faster than new/new trials, because preempting associations implies a restriction of the search space (Fig 1B), and thus less competition [40–42]. Of course, this latter effect might be negligible if the space of possible associations is vast. Thus, we also consider the possibility that proactive control will reduce RTs specifically in trials in which the number of possible associations is relatively small.

**Fig 2:**
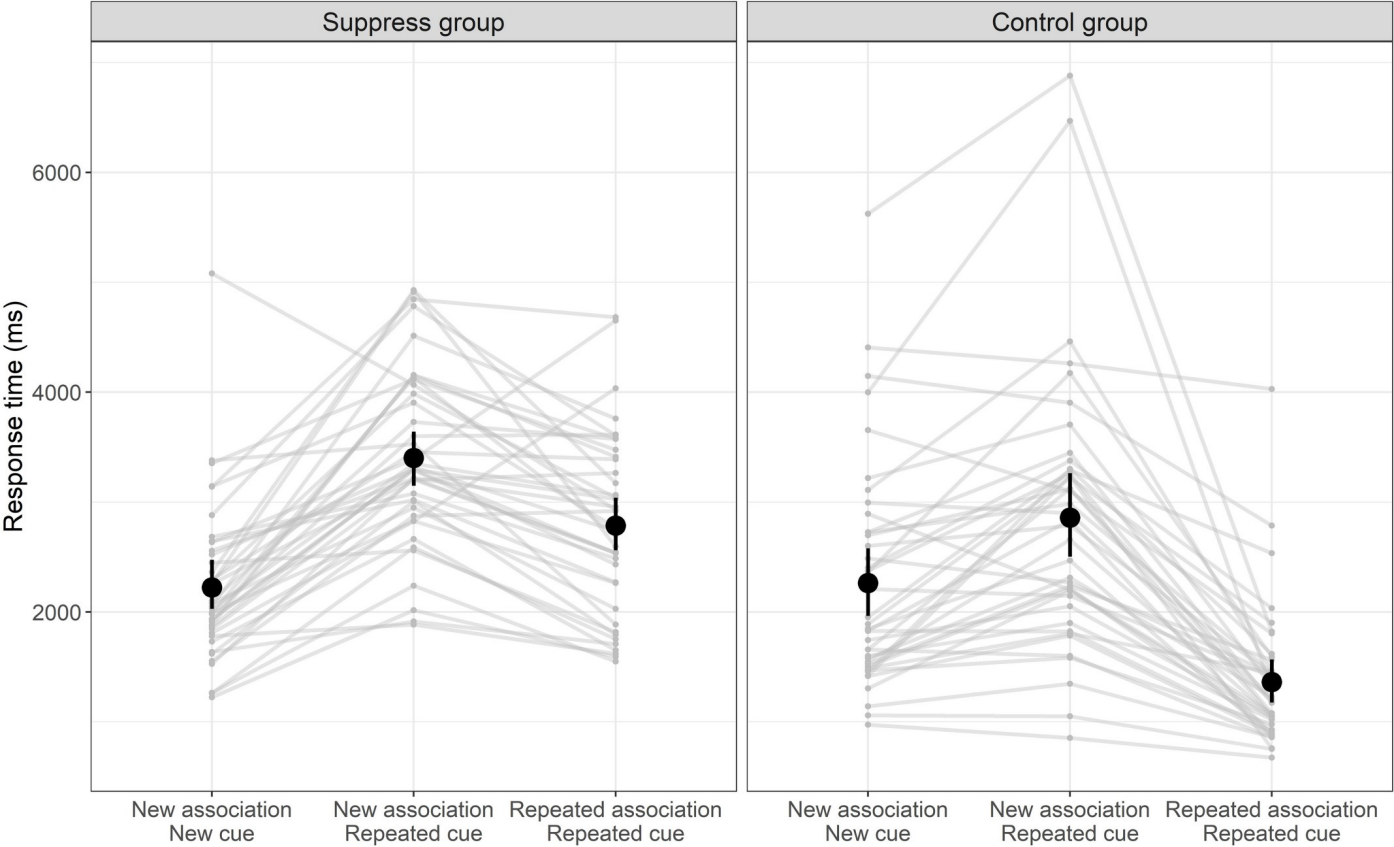
Median response times for the two groups, and different trial types. Black points represent average across participants (± 95% bootstrap confidence interval). Grey points and lines depict median response times for individual participants.

Comparing the difference in log RT to new and repeated cues for the two groups (Fig 2), we found that, as predicted by reactive thought control, the attempt to avoid repeated associations resulted in a larger increase in average log RT (*t* (77.99) = 4.44, *p* < .001, Cohen’s *d* = 0.99). A similar effect was found comparing median raw RTs (*t* (77.72) = 3.51, *p* < .001, Cohen’s *d* = 0.79). Deceleration in the suppress group was most pronounced for cues with a minimal number of possible associations (as indicated by previous norms [57]; *β*_intercept_ = 0.61, *SE* = 0.05, *Z* = 11.87, *p* < .001; linear mixed-effects model), and less so for cues with more possible associations (*β*_slope_ = -0.02, *SE* = 0.003, *Z* = -7.42, *p* < .001). This result is consistent with reactive control because when the number of possible associations is small, associations are more likely to recur and require rejection. Additionally, this result rules out the possibility of a proactive control that is restricted to cues with few predictable associations,

One difficulty deducing an absence of proactive thought control from these results is that if previous associations were particularly strong, even proactive thought control might predict slower responses, because excluding the strongest associations from the search space may paradoxically lead to a less lopsided, and therefore more protracted, competition between many equally weak associations. A more specific prediction of proactive thought control is thus that new/repeated trials will be faster than new/new trials mainly when previous associations given to this cue were weak, since eliminating such associations conclusively reduces competition.

To examine this prediction, we used a linear mixed-effects model to regress the difference in log RT comparing new/repeated with new/new trials (for each participant and cue) on the average strength of the participant’s previous associations to the cue. For the case in which previous associations were weakest, proactive thought control unequivocally predicts faster responses, whereas reactive thought control predicts slower responses if weak associations are likely enough to be resampled and rejected, and no change if weak associations are not likely to be resampled. This analysis showed that even when previous associations given to a particular cue were weakest, the attempt to avoid repeating them increased log RT (*β*_intercept_ = 0.15, *SE* = 0.04, *Z* = 4.16, *p* < .001), as consistent with reactive control.

The above analyses strongly suggest that people mostly employ reactive control to regulate specific unwanted associations. However, they might still be using three latent types of proactive control: (a) reducing the natural episodic increase in associative strength caused by previous retrievals of an association (Fig 1C); (b) preempting *all* repeated associations once one repeated association is sampled and rejected (Fig 1D); and (c) preempting only the rejected association from being immediately re-sampled (Fig 1E). We test for these more nuanced forms of control using an SMP model formalizing the involvement of different reactive and proactive processes underlying the selection of associations.

### Can people reduce the strength of repeated associations?

As a first step, we determined whether analyzing participants’ data using the SMP model corroborates the employment of reactive control to suppress repeating associations. For this purpose, we compared a model with no rejections (*α* = 0) to a model wherein repeated associations were rejected with probability *α*. The results showed that participants in the suppress group exhibited a strong tendency to reject repeated associations (with an average probability, formalized by the α parameter, of 0.7), whereas no evidence for rejection was found in the control group (Table 1). Furthermore, the effect size of this group difference, measured by allowing α to be freely estimated in both groups (thus, using the non-optimal model in the control group) was large (*t* (73.87) = 13.09, *p* < .001, *d* = 2.93), with only a minority of participants in the control group appearing to reject some repeated associations (17.5% of control participants had an α value above 0.05, and only 3 control participants had a value above 0.5).

Next, we examined whether participants in the suppress group were able to preempt all repeated associations after the first repeated association that came to mind was rejected (Fig 1D). The data did not support a model formalizing this process (Table 1), wherein another parameter (*ρ*_w_) was added to control the modification of associative strength after a rejection. Indeed, the best-fitted values for this additional parameter were positive (*M* = 1.01, *t*(39) = 2.24, *p* = 0.031, *d* = 0.35), and did not differ significantly from the standard associative strength modification parameter *ρ*, controlling the weakening/strengthening of the first repeated association (*t* (39) = 1.00, *p* = 0.32, *d* = 0.16). Conversely, a model in which rejected associations were not allowed to be immediately resampled (Fig 1E) was supported by the model comparison results (Table 1), suggesting that participants in the suppress group were able to avoid getting stuck in a loop in which the same repeated association is repeatedly generated and rejected.

Importantly, parameter recovery for the best-fitting models was adequate in that each recovered parameter correlated more strongly with its original fitted value than with the values of the other fitted parameters. Furthermore, evidence for the validity of the model comparison results was obtained by simulating data from each model and testing the relative fit of the different models in explaining this simulated data (see Figs G-L in S3 Text).

**Table 1:** Model comparison results. The table shows Bayesian Information Criteria Difference in fitting the experimental data from the two study groups, relative to the model that best fitted each group (for which the difference is 0).

Model	Suppress group	Control group
No rejections	226.8	0
With rejections	93.6	151.0
With rejections + post-rejection preemption	240.5	268.7
With rejections + no resampling of rejected association	0	149.5

### Can people reduce the episodic increase in the strength of repeated associations?

Consistent with a rehearsal effect, we found that generating an association in response to a cue increased its associative strength in both control (*M* = 3.07, *SD* = 1.09) and suppress (*M* = 1.41, *SD* = 0.54) groups, as indicated by the positive value with which the associative strength modification parameter (*ρ*) was best-fitted. In fact, all individually fitted *ρ* values were above zero. The implied episodic strengthening of previously reported associations explains why avoiding even weak prior associations (in the suppress group) delays responses (as reported in the mixed-effects model results above). Specifically, if generating an association does not increase its strength, then the probability that weak associations will recur is extremely low, and thus reactive control is unlikely to be needed. Conversely, an episodic increase in the associative strength of repeated associations can make even weak association likely to recur, and thus get rejected.

Importantly, however, although repeated associations became stronger in the suppress group, this increase was considerably lower than the increase found in the control group (*t* (57.29) = -8.54, *p* < .001, *d* = -1.91), suggesting that people were able to partially mitigate the natural increase in the strength of previously reported associations (Fig 1C). This effect should also manifest in slower (inadvertent) reporting of a repeated association in the suppress group, particularly in trials in which the cue repeats for the first time, since in these trials, slower responses cannot be explained by preceding rejection of other repeated associations. Indeed, the repeated/repeated–new/new log RT difference in these trials was larger in the suppress group (*t* (64.96) = 5.83, *p* < .001, *d* = 1.32).

### How well does the model explain the data?

**Fig 3:**
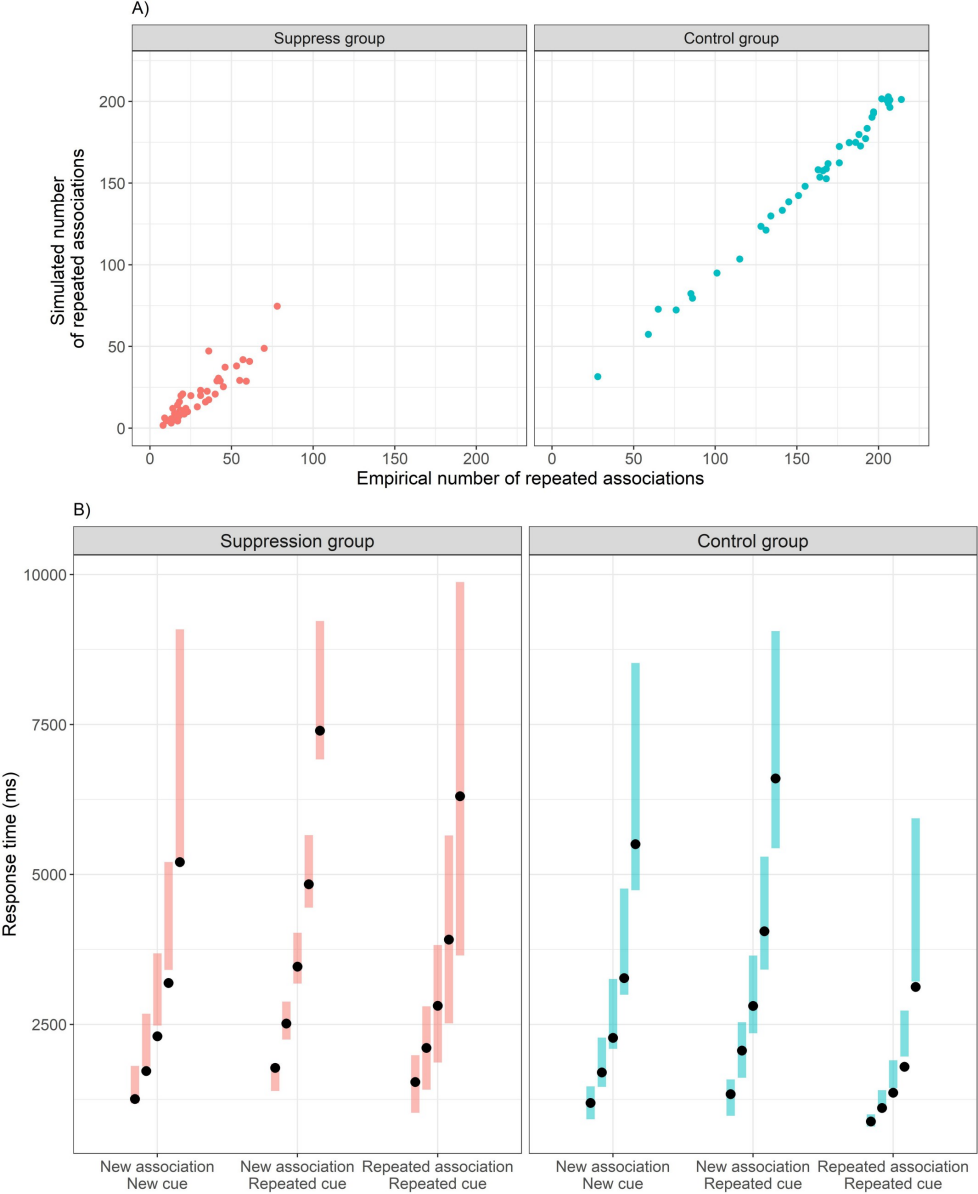
Absolute fit of the winning SMP models (see Table 1) in both groups. A) simulated vs. empirical number of repeated associations per participant. B) simulated (error bars) vs. empirical (black points) RT distributions (10^th^, 30^th^, 50^th^, 70, and 90^th^ quantiles are depicted) for the different trial types, averaged across participants.

To determine whether the modeling successfully captures essential properties of participants’ associations and RTs, we examined the correspondence between participants’ real data and data simulated using each group’s best-fitting model (Table 1) with parameters set to the values that best fitted participants’ data. We found that the models successfully captured group differences and individual differences in the number of repeated associations (Fig 3A). The models also successfully captured most RT quantiles, as well as key group RT effects–slower RTs for repeated/repeated and new/repeated trials in the suppress group in comparison to the control group (Fig 3B). That said, the model tended to slightly overestimate high RT quantiles in repeated/repeated trials in the control group. Furthermore, for new/new trials, the simulated but not the empirical data demonstrated slower performance in the suppress group. Both misfits are likely related to the fact that these quantiles were relatively rare (reflected in the width of the error bars in Fig 3B), and thus the model may not account for them well enough.

Finally, we also found group differences in some of the other model parameters that control RT (|*t’s|* >6.4, *p*’s < .001, |*d*’s| > 1.43; for further details see S2 Fig). However, these unpredicted effects were smaller than the effects found for the rejection parameter, *α* (2.93) and the associative strength modification parameter, *ρ* (-1.91), suggesting that the latter represent the key cognitive processes distinguishing the groups. Some of these differences might reflect a global effect of task instructions on response style or strategy (e.g., extra caution that participants in the suppress group generally took while encoding cues or responding), or time-consuming processes involved in deciding whether a particular association had already been reported. Importantly, we conducted a sensitivity analysis to verify that our key modeling findings (e.g., group difference in *α* and *ρ*) are not contingent on the group differences in other RT-controlling parameters. In keeping with the between-subject design and the fact that each individual was fitted separately, we performed the sensitivity analysis by re-fitting the thought suppression parameters while holding the values of the other RT-controlling parameters fixed to the control group mean (fixing to the suppress group mean produced similar results). The results of this analysis replicated the main findings reported above, in that *α* values were considerably greater in the suppress group (P(α_control_ < 0.001) = 75.0%*; t* (53.75) = 13.80, *p* < .001; *d* = 3.09), whereas *ρ* values were significantly lower in the suppress group (*t* (68.05) = -3.73, *p* < .001; *d* = -0.83), but still higher than 0 for all participants in that group (range = [0.32, 2.20]). Furthermore, the no-resampling model (Fig 1E) performed better than the model allowing re-sampling of rejected associations (Fig 1A) in the suppress group (ΔBIC = -63.2). Thus, the main results concerning group differences in the parameters directly affecting thought control did not depend on group differences in other RT-related parameters.

### Exploratory analysis of additional control processes

Whereas the present experiment was designed to test how participants control repeated associations, participants were also asked to report only strong and common associations. This was necessary to ensure that participants in the suppress group do not adopt non-associative strategies to avoid repetitions (e.g., reporting different objects they see in the room, regardless of the cue). However, it is not unreasonable to assume that this instruction motivated an additional control process (associative strength control; *AS control*) so as to report stronger associations. To test whether such AS control was employed, and whether it interacted with the control over repeated associations, we enhanced the model with proactive and reactive AS control mechanisms. As delineated in S5 Text, proactive AS control was formalized using a non-linear transformation changing the variance of the AS distribution (e.g., making strong associations even stronger). Conversely, reactive AS control was formalized by allowing the model to reject (repeated or new) associations with a probability that is inversely related to their strength. This analysis was deemed exploratory because no relevant hypothesis was preregistered, nor did we have a suitable control group in which no emphasis on strong associations was given.

Model comparison results (Table A in S5 Text) supported proactive AS control, with less support for reactive AS control. Crucially, our main findings held with this enhancement of the model, in that only the suppress group tended to reject repeated associations (${\bar{\alpha }}_{suppress}=.69,\;{\bar{\alpha }}_{control}=.08;$ *t* (73.75) = 12.32, *p* < .001, *d* = 2.75, with stronger evidence for a model wherein α was fixed to zero in the control group), and mitigated the natural increase in the strength of repeated associations (${\bar{\rho }}_{suppress}=1.08,\;{\bar{\rho }}_{control}=2.88;$ *t* (73.65) = 7.15, *p* < .001, *d* = 1.60). Finally, some evidence for a tradeoff between the two targets of control was found, in that the degree of AS control was lower in the suppress group than in the control group (*t* (74.30) = 2.9, *p* < .005, *d* = 0.66). We discuss the implication of these intriguing, yet exploratory results in the discussion below.

### Exploratory analysis of individual differences

Finally, we asked whether thought control measured by the experimental task covaried with how participants experience thought control in their daily lives. For this purpose, we examined participants’ scores on the Effective suppression subscale of the Thought Suppression Inventory–Revised (TSI-R-effSup) [59], which measures the extent to which people experience their daily thought control attempts as effective. The results suggested that self-reported effective suppression in daily life was predicted by the thought rejection parameter (*r* (38) = 0.33, *p* = .03) in the suppress group. Thus, people who were more successful in rejecting unwanted associations that came to mind, also reported themselves being more successful in controlling unwanted thoughts in their daily life. Furthermore, a significant correlation was found between the associative strength modification parameter (*ρ*) and self-reported effective suppression in the control group (*r* (38) = -0.32, *p* = .045). This result suggests that self-reported thought control efficiency is associated with a weaker enhancement of previously generated associations. The correlations between these task-related variables and other scales measuring similar constructs were in the same direction but weaker (see S3 Fig). These results should be taken with caution since they were not precisely predicted. However, two important implications may be considered. First, these results demonstrate the potential of our task and model for investigating the cognitive processes that distinguish people who can control negative thoughts from those who struggle in this regard. Second, the latter result coheres with the idea that the everyday experience of unwanted thoughts escalates because having a thought increases the probability that it will be experienced again. In this way, unwanted thoughts might develop into a form of mental habit [66]

## Discussion

People have different motivations to control their thoughts. Some thoughts might be unpleasant, whereas others might simply be distracting or unproductive. Here we examined the extent to which people deal with thoughts that are inconsistent with current goals by proactively reducing their ’volume’ such that they will not come to mind. This possibility has been inspired by the intentional forgetting literature, suggesting that people can successfully weaken artificial associations learned in the lab, without requiring extensive ’suppression practice’ [21,23,24]. Our results indicated that people mostly engage in reactive thought control, stopping unwanted thoughts after they occur. However, proactive thought control played two key roles.

First, when asked to suppress repeated associations, participants could mitigate the temporary increase in associative strength that characterizes previously generated associations. This conclusion was supported by both raw behavioral analysis and modeling, with the latter suggesting that this form of proactive control is weaker than reactive control but is still very strong. Crucially, participants did not manage to reduce associative strength below its long-term baseline, nor were they able to completely eliminate the increase in associative strength of repeated associations. This finding qualifies previously reported findings regarding human’s ability for intentional forgetting (in the think-no-think and directed forgetting paradigms [21,23,26,27,67]). Understandably, these previous experimental approaches have not been used to investigate long-term associations, likely due to the infeasibility of making people entirely forget such associations. The novel paradigm and model presented here provide a more sensitive measure for proactive control over long-term memories, by measuring (covert and overt) spontaneous recall. Whereas our results demonstrate proactive control over a potential strengthening of long-term memories, intentional weakening of such memories seems to be impossible.

Second, although people are destined to experience an unwanted thought before being able to stop it, our findings suggest that stopping a thought after it appears also prevents it from immediately coming to mind again, allowing people (in the suppress group) to replace the rejected thought instead of remaining in an endless loop. This form of proactive control may reflect the high predictability of the rejected association, and could be implemented similarly to the gating of repeatedly presented external stimuli [9,44,45]. Importantly, not all sorts of predictability enabled proactive control. Modeling the present data suggested that being able to predict a cue that can evoke different unwanted associations did not allow participants to preempt these associations. This points to the intriguing idea that our own thoughts remain unpredictable to us, even when constrained by a specific context. We note though that since the conclusions concerning the effects of predictability were only deduced via modeling, they should be taken with greater caution, and augmented by future studies using other measures of latent thought processes (e.g., neural decoding). Furthermore, future studies could examine whether the gating effect we found only affects the next sampled association, or whether this effect persists until an association is reported.

Together, these findings highlight the shared and unique characteristics of thought control. Whereas predictability increases the likelihood for proactive control for both external [11,12,14,38,68] and internal stimuli, such predictability appears to be much harder to ascertain for associations, because the number of options is much larger and changes across people and cues. Our results indicate that an association is predictable enough only if it came to mind just a moment ago. Future studies could examine whether proactive control over free associations is made possible if participants are instructed to avoid a limited number of associations that are common to many cues, or certain well-defined groups of associations (e.g., avoid only weak associations / avoid taxonomic associations such as dog-cat vs. dog-bark). Indeed, exploratory analysis suggested that our participants may have been able to enact proactive control to report stronger association, which presumably entails strengthening a group of (already strong) associations (S5 Text). Future studies could test this explanation with proper control conditions.

### Implications for the study of sequential memory retrieval in other contexts

Reactive and proactive control are also indirectly implicated in previous work examining semantic retrieval or implementing such retrieval mechanisms as part of a more complicated task. For example, a previous model of the semantic fluency task assumes that people traverse semantic memory in a random walk while visiting, but not reporting repeated words [48,69]. Such reactive control is crucial for this model’s ability to account for the finding that people’s responses slow down with each additional instance until they switch to another sub-category [70,71]. A competing, influential model has attributed these slowdowns to the depletion of ’semantic resources’ in a specific sub-category, leading to a strategic switch to a different category [60,72], although whether people’s sensitivity to such depletion involves reactive or proactive control is unknown. Critically, to our knowledge, these models have not been previously fitted to reaction time data, and inter-item response times are usually assumed to be constant and independent of associative strength. The semi-Markov process model developed here could provide a tractable solution for explaining word choices and inter-word intervals as an interaction between associative strength, reactive and proactive control, and strategic foraging.

Importantly, sequential memory retrieval processes likely play a key role in many other, more complex tasks [49–51,73]. For example, a Markovian random-walk model over semantic memory has recently been used to formalize the process through which people retrieve response alternatives in an open-ended decision-making task [50]. The probability of producing a particular response in this task was found to be governed by an interaction between the degree of preference for the response option and the probability of retrieving a sequence of options that includes it. Interestingly, the authors have also found that, is some cases, retrieving an option earlier positively biased its evaluation, an effect also shown in another theory of value construction in a different context [73]. However, whether such primacy indeed biases value directly [50], or is mediated through an effect on memory [73] is yet unclear, since the possibility that early retrieval would augment subsequent retrieval (as found in our study, and in [73]) was not considered. One intriguing prediction stemming from our results is that if indeed the primacy effect reflects memory rather than value boost, the question of whether participants are asked to avoid or ignore repeated recollections of options will moderate this primacy effect. More generally, these previous works have only examined participants’ choices and could thus be meaningfully enhanced by also analyzing reaction times using the SMP model developed here. Indeed, such joint choice-RT analysis is likely to be informative for any task that is thought to involve a sequential retrieval process.

### Implications for the study of individual differences in thought processes and creativity

The experience of uncontrollable, unwanted thoughts is strongly implicated in many psychiatric disorders [2,74,75], yet studies of clinical populations measuring actual (as opposed to self-reported) control over thoughts and memories have produced mixed results [24,76,77]. The current work can advance the understanding of how thought control is impaired in clinical populations by focusing on the latent, computational mechanisms that enable it. Indeed, despite the recent growth in the use of computational modeling to characterize the mechanisms that give rise to mental illness [78–80], computational models of thought processes and their control are scarce in psychiatry.

Our preliminary results in this regard suggested two distinct mechanisms responsible for self-reported difficulties in thought control. Thus, whereas self-reported thought control efficiency was related to a lower tendency to repeat associations, this was explained by a reduced enhancement of repeated associations in the control group, and their increased rejection in the suppress group. Indeed, the behavioral effects of the latter were quite pronounced as some individuals reported a repeated association in more than 50 (20%) trials. Whereas our model identified this as a deficit in reactive control, it does not explain what exactly caused a failure to reject repeated associations. One possibility is that such failure reflects a general difficulty in inhibiting predominant responses. Indeed, previous studies have suggested that the tendency of some psychiatric populations to experience intrusive thoughts is related to a general inhibition failure [81,82]. This is consistent with the intriguing possibility that stable individual differences in proactive and reactive control of externally directed attention [11,12] also manifest in the control of internally generated thought. However, despite the appeal of this interpretation, we cannot rule out the involvement of other factors in the inhibition failure found in some individuals in the suppress group, including a lack of motivation, or a difficulty remembering whether an association has been reported before or not. Interestingly, this latter, putative mechanism is reminiscent of findings linking intrusive thoughts (and compulsive checking) to a difficulty in remembering whether a certain action was performed or imagined [83,84].

The finding that individual differences in our non-emotional task were associated with a self-reported tendency to experience unwanted thoughts in daily life suggests the involvement of a general, valence-independent memory control mechanism. In this, our findings cohere with two recent studies suggesting the operation of valence-independent mechanisms in intrusive thought [5,7]. Future studies could examine how the affective significance of cues and associations, as measured for instance by valence and arousal ratings, modulate our group-level and individual-differences results.

More specific impairments in semantic retrieval and semantic control characterize other psychiatric [85–87] and neurological conditions [35,88,89]. Whereas in some of these cases an impairment in avoiding the repetition of responses is observed (i.e., in cognitive impairment [89,90]), other conditions (i.e., schizophrenia, semantic aphasia) involve difficulties in inhibiting different types of predominant [35,91] or overly loose associations [85,92]. The framework developed here can be adapted to examine how semantic representation interacts with proactive and reactive semantic control in affected individuals. A straightforward example of how this could be achieved is provided by our extended, exploratory model examining how participants in our task attempted to provide relatively strong associations (see S5 Text).

The ability to generate unique associations has also been implicated in creativity research [51,93–96]. However, it is yet unclear whether creative individuals generate more unique, distant associations because their associative maps are differently structured [97,98], or due to a better executive ability to inhibit predominant associations [52,99,100]. Whereas most previous creativity research measured control processes using separate executive function tasks (e.g., Stroop; [101]), the SMP can be easily extended to study the interaction between semantic structure and control within the same task. Thus, for example, by asking a group of participants to produce creative associations, one can examine whether more creative individuals (as measured by an independent task) differ in associative structure (in which case more creative associations are also expected without these instructions), in proactive control (allowing them to modify their associative structure in accordance with the instructions), or in reactive control (allowing them to more effectively reject predominant associations).

### Limitations and conclusion

In contrast to previous paradigms [23,24,102], we did not direct participants, nor were we able to incentivize them, to avoid *thinking* a particular thought. Rather, feedback and monetary bonus depended on participants’ reported responses. That being said, participants are required to think of non-repeated associations to write them, such that even covertly thinking of repeated associations is inconsistent with the task and thus leads to inefficient performance. More generally, this study was not designed to examine the limits of human thought control abilities but rather the spontaneous processes through which people minimize distracting, unwanted thoughts. This is in line with recent efforts focusing on the conditions and personal characteristics that make proactive control prevalent [11]. More generally, the modeling framework developed here can be used to advance the research of proactive and reactive control in multiple domains, by advancing analysis from the level of raw RTs to the latent processes explaining them.

## Supporting information

S1 Text Reasons for excluding specific participants. (DOCX)

S2 Text Additional details on stimuli preparation for the free association task. (DOCX)

S3 Text Additional details regarding the SMP.
**Fig A**. Illustration of the transition matrix governing the semi-Markov process used to model consecutive generation of associations in the task.
**Figs B-F**. Parameter recovery for alternative parameterizations of the SMP.
**Figs G-L**–The recovery of parameter values and model comparison results for the main SMP parameterizations used in the paper.
(DOCX)

S4 Text Specification of the method used to estimate covert spaces of associations.
**Fig A**. The distribution of normalized associative strength of different associations to the word Table, as extracted from human free association norms vs. and corpus-based cosine similarity.
**Fig B**. The association between the data and the predictions of the model estimating the space of associations with regards to whether an association is normed or not and its rating.
**Fig C**. Testing the sensitivity of the method we used to estimate the number and strength of non-normed associations to a different choice of bin-width.
(DOCX)

S5 Text Specification of an adapted SMP model with either proactive or reactive inhibition of weak (i.e., uncommon) associations.
**Table A**.–Supplementary model comparison results.
(DOCX)

S1 Fig Sensitivity analysis. (DOCX)

S2 Fig Best-fitted parameter values in the two groups. (DOCX)

S3 Fig Correlation matrix of the different self-report measures and the SMP model parameters. (DOCX)
