# Supplementary material for: If you don’t let it in, you don’t have to get it out: Thought preemption as a method to control unwanted thoughts
Source: PLoS Comput Biol. 2022 Jul 14;18(7):e1010285. doi: 10.1371/journal.pcbi.1010285 (PMC9282588; doi:10.1371/journal.pcbi.1010285)
Supplement: S1 Text — (DOCX) [file pcbi.1010285.s001.docx]

**S1 Text. Reasons for excluding specific participants**

| **Participants excluded due to failing two or more attention check types in the rating phase** | |
| --- | --- |
| 1 | Failed 3/3 explicit attention checks and had a very low pTP-rating correlation |
| 2 | Failed 3/3 explicit attention checks and had a relatively low pTP-rating correlation |
| 3 | Failed 1/3 explicit attention check; pTP-rating correlation is negative; Missed rating catch trials. |
| 4 | Failed 3/3 explicit attention checks; pTP-rating correlation is negative. |
| 5 | Very low pTP-rating correlation; Missed rating catch trials |
| 6 | Failed 2/3 explicit attention checks; Low pTP-rating correlation; Exited task screen twice. |
| 7 | Failed 3/3 explicit attention checks; Missed rating catch trials. |
| 8 | Failed 1/3 explicit attention checks; Low pTP-ratings correlation; Missed rating catch trials |
| 9 | Failed 3/3 explicit attention checks; Low pTP-rating. |
| 10 | Failed 3/3 explicit attention checks; Missed rating catch trials |
| 11 | Negative pTP-rating correlation; Missed rating catch trials |
| **Participants excluded due to time-out responses in >30% of trials** | |
| 12 | Timed out 98/300 trials. |
| 13 | Timed out 100/300 trials; Also negative pTP-rating correlation |
| **Participants excluded due to an exceptionally low variance in ratings** | |
| 14 | All ratings were 100; Also poor performance in ratings-phase catch trials |
| 15 | >90% of ratings are above 97. |
| 16 | >90% of ratings are above 97. |
| 17 | >90% of ratings are above 97. |
