# Supplementary material for: If you don’t let it in, you don’t have to get it out: Thought preemption as a method to control unwanted thoughts
Source: PLoS Comput Biol. 2022 Jul 14;18(7):e1010285. doi: 10.1371/journal.pcbi.1010285 (PMC9282588; doi:10.1371/journal.pcbi.1010285)
Supplement: S2 Text — (DOCX) [file pcbi.1010285.s002.docx]

**S2 Text. Additional details on stimuli preparation for the free association task**

Sixty cues were derived from Nelson [[1]](https://sciwheel.com/work/citation?ids=2936686&pre=&suf=&sa=1&dbf=0) We used only relatively familiar words (cues with high frequency, i.e., above the 70^th^ quantile of the distribution of cues in that study), consisting of 4 to 6 letters (to minimize variance in the time it takes to read the cue). Homographs were excluded. Since previous work has shown that the variance (i.e., entropy) of the distribution of how many different associations were given to a cue across participants is a crucial component in determining the amount of competition and thus speed, we opted to have a wide range of entropy. Thus, we pre-selected high and low entropy cues were pre-selected (using the 8^th^ and 93^rd^ entropy quantiles).

Cues for which the most prevalent association also appeared as the cue in another trial were excluded to minimize potential carryover effects of such cues. Next, cues that were a grammatical variation on existing cues (e.g., ‘Woman’ and ‘Women’) were excluded. Finally, cues with a clear negative or arousing connotation (e.g., ‘Death’,’ Bomb’) were excluded. This procedure resulted in 68 cues, 8 of which were used for a short training block, whereas the remaining 60 were replicated five times each and split into five equal-sized blocks. The order in which these cues were presented was completely random and differed across participants.

[**References**](https://sciwheel.com/work/bibliography)

[1.    Nelson DL, McEvoy CL, Schreiber TA. The University of South Florida free association, rhyme, and word fragment norms. Behav Res Methods Instrum Comput. 2004;36: 402–407.](https://sciwheel.com/work/bibliography/2936686)
