# Supplementary material for: If you don’t let it in, you don’t have to get it out: Thought preemption as a method to control unwanted thoughts
Source: PLoS Comput Biol. 2022 Jul 14;18(7):e1010285. doi: 10.1371/journal.pcbi.1010285 (PMC9282588; doi:10.1371/journal.pcbi.1010285)
Supplement: S3 Text — Additional details regarding the SMP. Fig A. Illustration of the transition matrix governing the semi-Markov process used to model consecutive generation of associations in the task. Figs B-F. Parameter recovery for alternative parameterizations of the SMP. Figs G-L–The recovery of parameter values and model comparison results for the main SMP parameterizations used in the paper. (DOCX) [file pcbi.1010285.s003.docx]

**S3 Text. Additional details regarding fitting the SMP**

As noted in the main text, the process in which candidate associations are generated successively, with varying speeds, until one association is accepted can be formulated as a semi-Markov process. The cue and each association constitute a state, and the process continues until an absorbing state – corresponding with an accepted association – is reached. To construct the semi-Markov process, two matrices are needed: (a) a matrix defining the probabilities of transitioning between states (e.g., from the cue, or a rejected association, to the next candidate association); (b) a matrix of parameters determining how long it takes to complete each transition. We provide a simple numerical example of this model given a cue with three possible associations, where p(A) ={0.5, 0.3, 0.2} and where A_2_ constitutes an association that has been generated before to that cue, such that p(reject) = {0, α, 0}. The transition probability matrix of the model (for the variant not allowing the consecutive resampling of rejected associations) is depicted in Fig A in S3 Text. In this example, the first association to be generated is A_2_, an event that takes μ_2_ time, on average. Since it is a repeated association, it can be rejected (with probability α). Only associations A1 and A3 can be generated in the following step since, in this specific model, rejected associations cannot be resampled in the consecutive time-step. This is formalized by the zeros on the diagonals of the upper left and upper right portions of the matrix (e.g., the transition from A_2_ ∩ Rej to A_2_ ∩ Rej), and the re-normalization of the remaining associations (e.g., the probability for the transition between A_2_ ∩ Rej to A_1_ ∩ Rej is now 0.71 rather than 0.5). In the model in which rejected associations may be consecutively resampled, all rows are similar to the first row, since in that model the probability to reach an association is not affected by the preceding association. In this specific example, the next association to be generated is A_1_, which takes μ_1_ time, on average. Since this is a new association, it is always accepted.


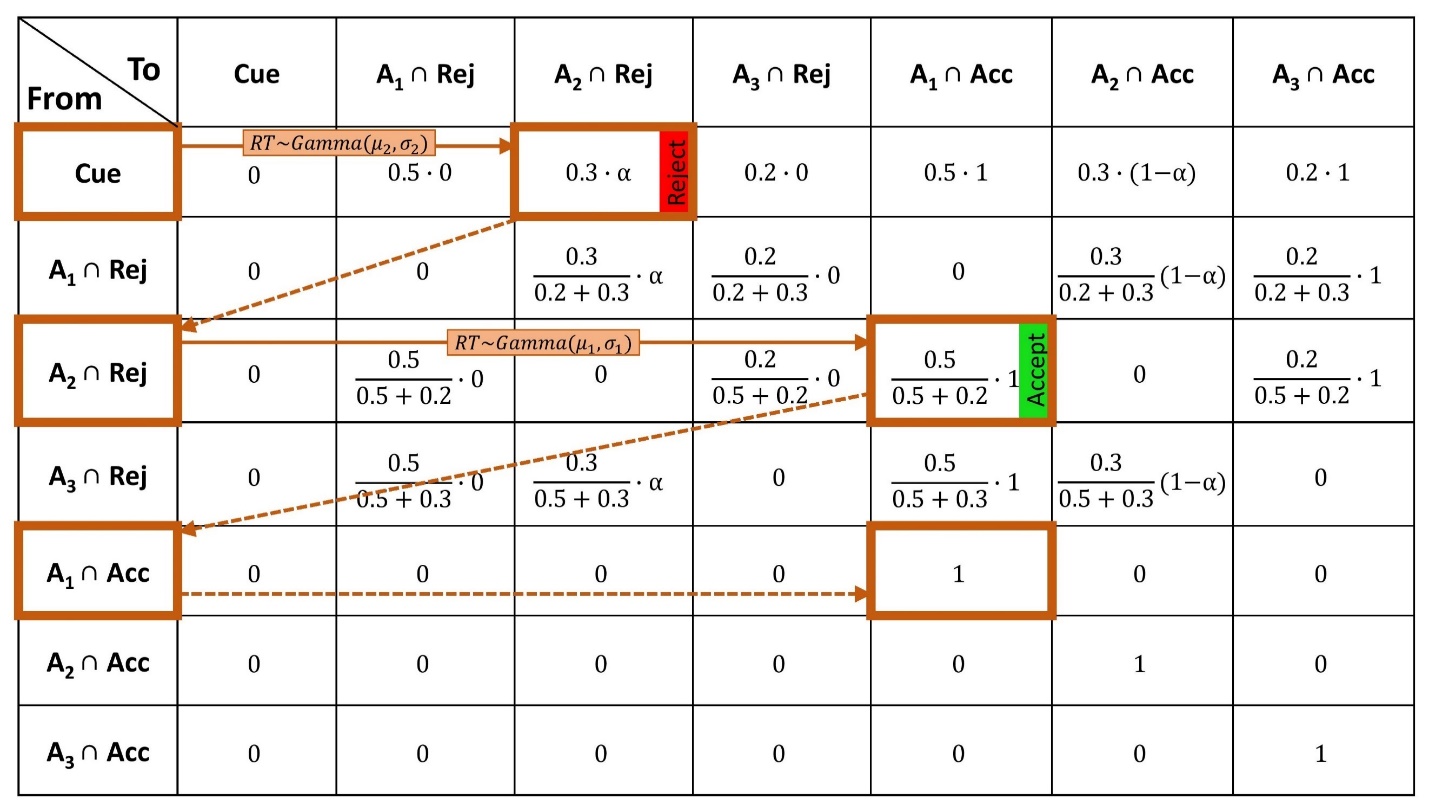


Fig A in S3 Text – Illustration of the transition matrix governing the semi-Markov process used to model consecutive generation of associations in the task. In this example, the participant first thinks of the second out of three associations (A_2_; the probability of which is 0.3), but rejects it with probability α. In the model depicted here, we assume that after an association is rejected, it cannot be immediately resampled (the probability of transitioning from A_2_ ∩ Rej to either A_2_ ∩ Rej or A_2_ ∩ Acc is zero). Thus, after rejecting A2, the participant can only think of A_1_ or A_3_ (such that their sum is now 1). We assume that both A_1_ and A_3_ are new associations that cannot be rejected (A_1_ ∩ Rej = A_3_ ∩ Rej = 0). In this example, the participant thinks of association A_1_ and accepts it. The periods of time it takes the participant to generate A_2_ and then A_1_ are determined by their associative strength and are parameterized using Gamma distributions. Dashed lines correspond with pseudo-transitions that do not take time.

The waiting time matrix consists of the μ_i_ and σ_i_ values corresponding to each association and is the same for rejection and acceptance states. These matrices are used in a well-established approximate method for solving SMPs using Laplace transformation to derive the distribution of the time it takes to accept a specific association (i.e., first-passage time distribution) [[1]](https://sciwheel.com/work/citation?ids=10645076&pre=&suf=&sa=0&dbf=0). This allows us to obtain the probability density that a process starting from the cue (state 0 in the matrix above) ends at accepting one of the three associations at time *t*.

Due to the extensive size of the estimated spaces of possible associations, the ensuing size of the transition probability and waiting time matrices of the SMP increased computation time to a level that made parameter fitting unfeasible (e.g., a single evaluation of the likelihood function can take up to 7 seconds, despite the use of efficient, parallelized code). Thus, we used an approximation to reduce the size of these matrices by aggregating states. In the case in which two or more associations have an equal p(A), the overall probability of transitioning to any of them is just the sum of their probabilities, and this aggregate’s μ is the same as the individual μ_i_ which are equal. Where probabilities are not equal but are similar enough, estimating an aggregate’s μ based on the mean of the individual μ_i_'s should result in minimal approximation error. Thus, after arranging p(A) values in order, we aggregated groups of p(A) with a maximal consecutive difference smaller than 0.005 (reducing the evaluation time of the likelihood function to ~180ms). Repeated associations were never aggregated. Note that all simulations used for parameter recovery analyses were based on the non-aggregated matrices, such that any estimation errors caused by this aggregation procedure are accounted for in the parameter recovery results. Yet, we recommend that researchers interested in comparing the SMP to other models either use similar aggregation for the other models, or use this aggregation for parameter fitting but remove it when evaluating the final deviance of the SMP.

The data were fitted using the Rmalschains R package [[2]](https://sciwheel.com/work/citation?ids=10645173&pre=&suf=&sa=0), which combines global-optimization (using an evolutionary algorithm), with gold-standard local search algorithms (Solis-Wets algorithm). These procedures helped avoid local minima while obtaining more accurate best-fit parameter values.

**Additional parameterization of the SMP**

We tested several additional parameterizations of RTs with respect to parameter recovery and absolute fit. First, we tested the performance of models where E^(μ)^ was fixed to 1 (indicating a linear relationship between $-\log\left[ {p(A}_{i} \right)]$and the mean of the Gamma distribution), and $\sigma$ was not a function of $\mu_{i}$, but rather a single value per participant.

A linear relationship between $-\log\left[ {p(A}_{i} \right)]$and the mean of the Gamma distribution allows to add an additional intercept parameter (I^(μ)^), replacing Equation 4 with:

$$\begin{aligned} \mu_{i}=I^{\mu}+e^{\boldsymbol{S}^{\boldsymbol{(\mu)}}}\cdot{(-\log\left[ {p(A}_{i} \right)])} \#A \end{aligned}$$

However, this model (Model S1) produced strong cross-correlations between parameters, particularly in the control group (see Fig B in S3 Text), and was thus not examined further:


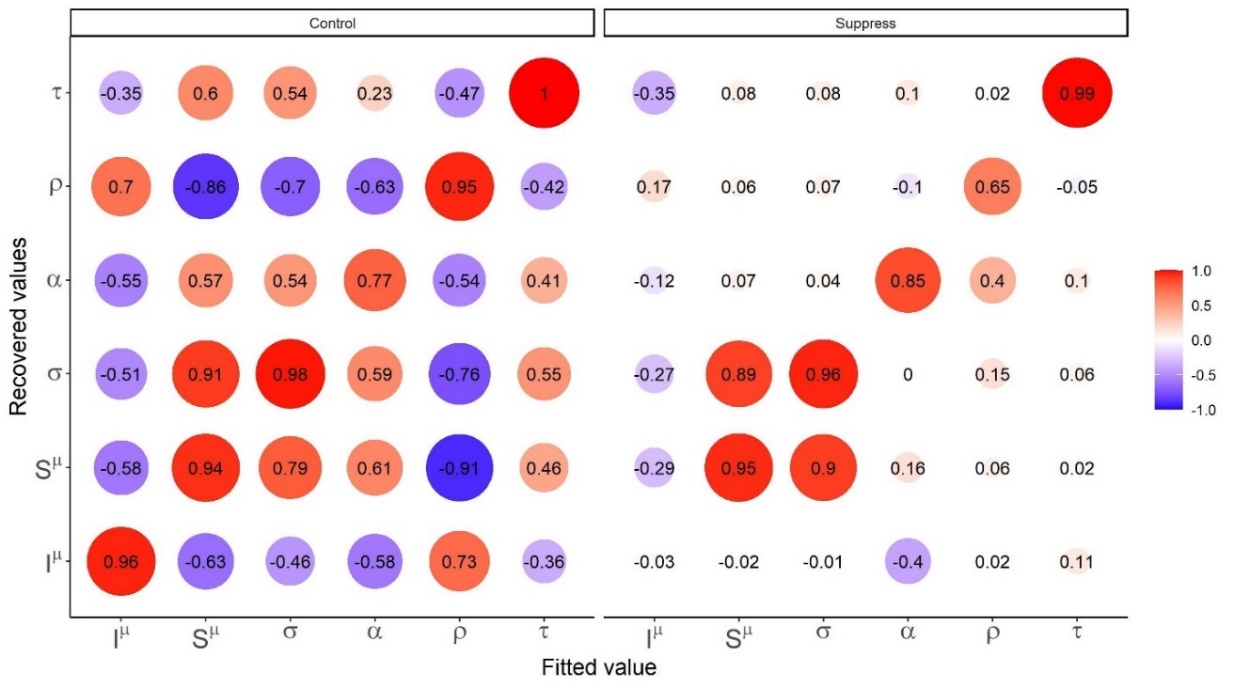


Fig B in S3 Text - Parameter recovery for Model S1 (S^(μ)^ fixed to 1, I^(μ)^ is free, σ is parameterized as a single number, not related to the mean of the Gamma distribution)

Fixing $I^{\mu}$ to 0 (Model S2) improved parameter recovery (Fig C in S3 Text), although a strong correlation remained between σ and S^(μ)^. More importantly, it produced a gross underestimation of the number of repeated associations in the control group (*M_model S2_* = 78.57 *M_data_* = 157.5).


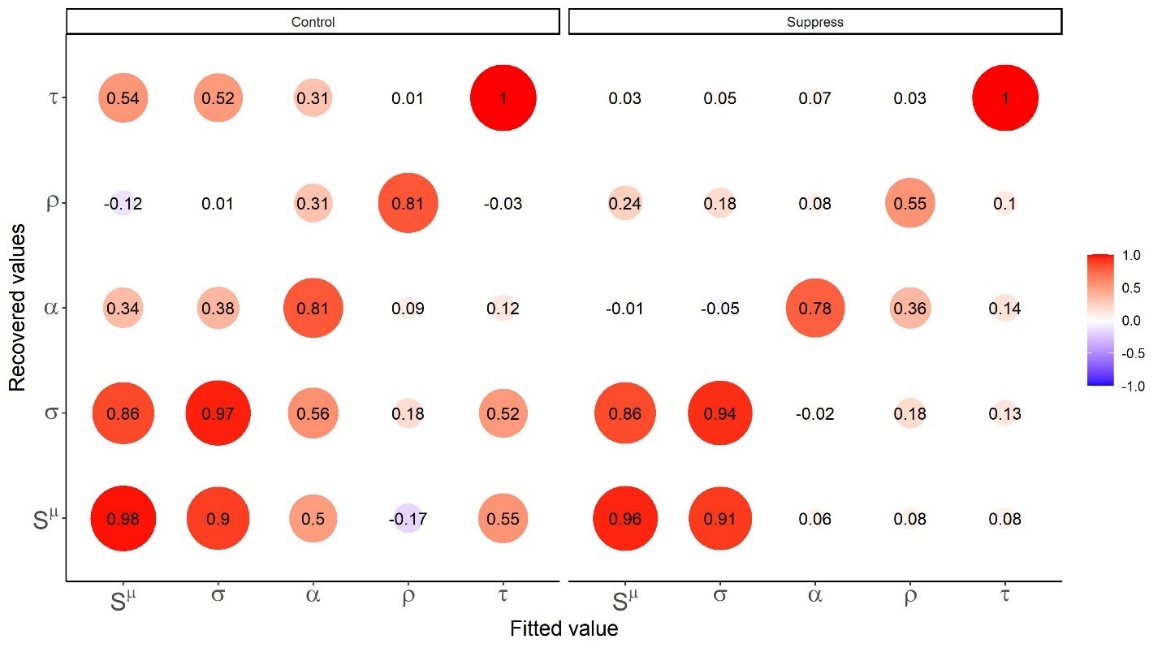


Fig C in S3 Text - Parameter recovery for Model S2 (S^(μ)^ fixed to 1, I^(μ)^ is fixed to 0, σ is parameterized as a single number, not related to the mean of the Gamma distribution).

A variation of this model, where $\sigma$ is a function of $\mu_{i}$ (as in the main text) produced poor parameter recovery (see Fig D in S3 Text)


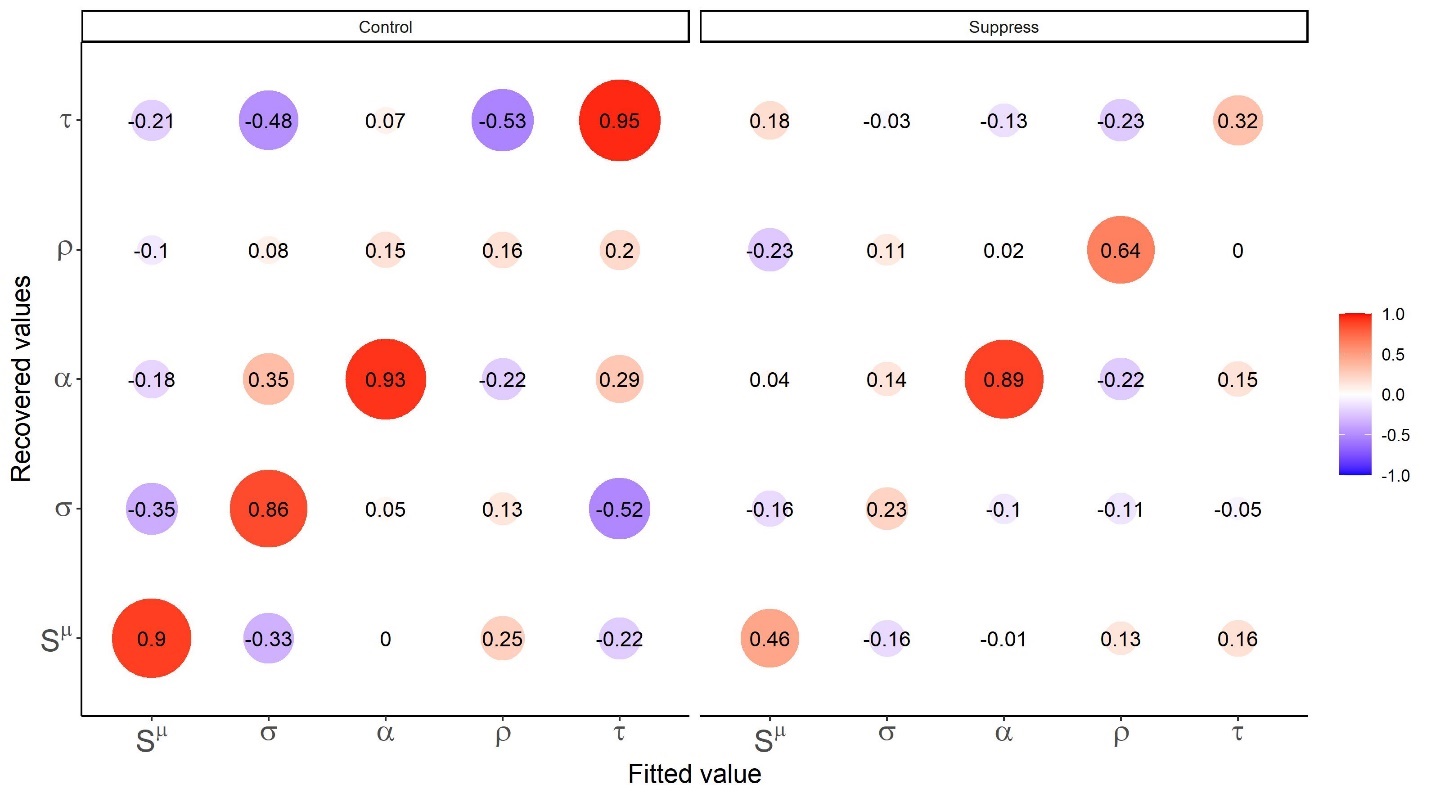


Fig D in S3 Text - parameter recovery for Model S3 (S^(μ)^ fixed to 1, I^(μ)^ is fixed to 0, σ is parameterized as a function of the mean of the Gamma distribution).

We also examined variations of the above models, wherein $-\log\left[ {p(A}_{i} \right)]$ controlled the rate of the gamma distribution, whereas the shape of the distribution was given by a single value per participant (Model S4). This model produces poor parameter recovery and was not explored further (Fig E in S3 Text):


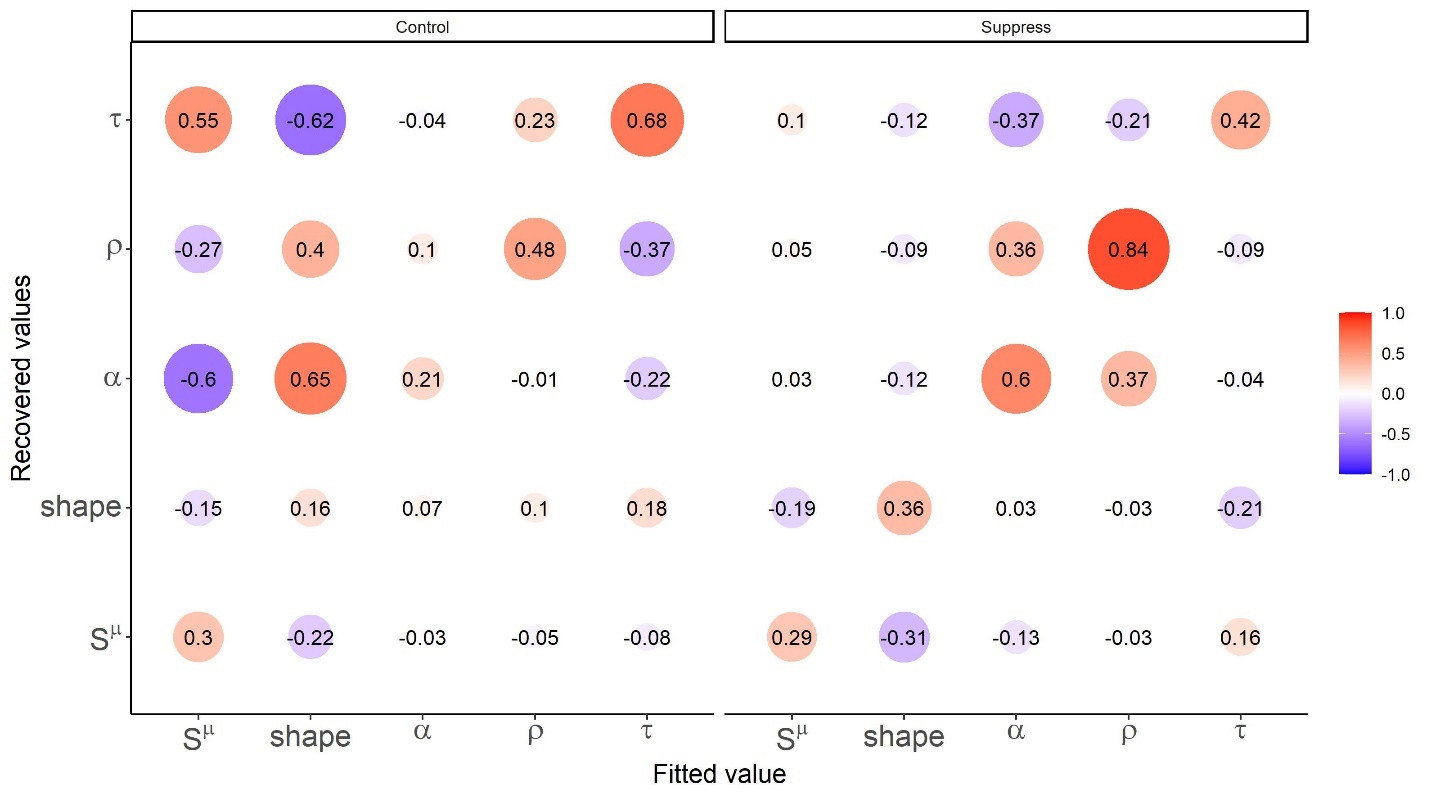


Fig E in S3 Text - parameter recovery for Model S4 (-log[P(A)] determined the rate of the Gamma distribution instead of its mean, I^(μ)^ is fixed to 0).

Finally, we examined a model where E^(μ)^ was free but $\sigma$ was not a function of $\mu_{i}$ (Model S5). This model showed cross-correlations above 0.65, and some auto-correlations that were below 0.65 (Fig F in S3 Text).
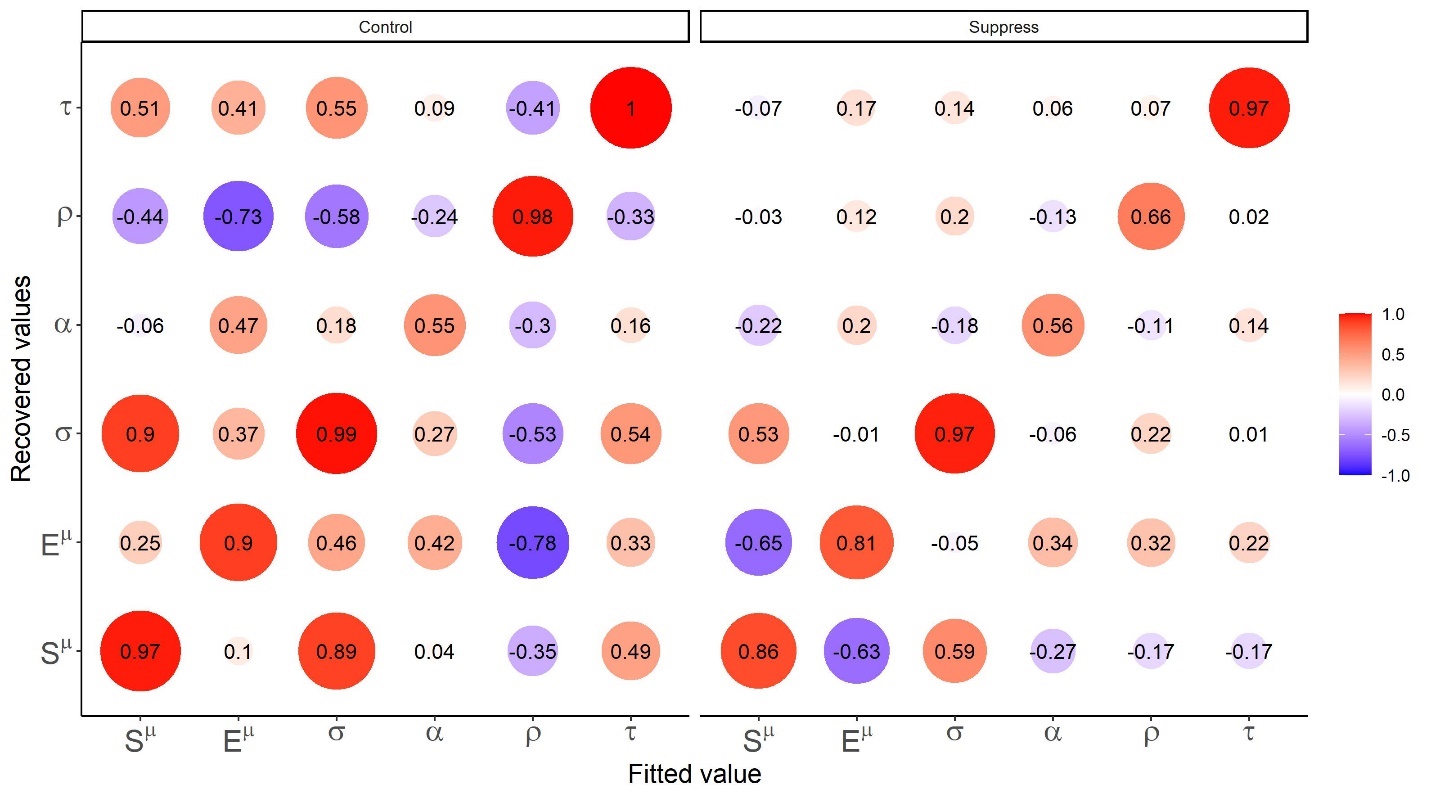


Fig F in S3 Text – Parameter recovery for Model S5 (S^(μ)^ is free, I^(μ)^ is fixed to 0, σ is parameterized as a single number, not related to the mean of the Gamma distribution)

Thus, the only model the produced reasonable parameter recovery was Model S2. As noted above, we did not use it because it underestimated the number of repeated associations, particularly in the control group. This also means that *ρ* was underestimated at that group. Nonetheless, this model has successfully replicated the group difference in *α* (*t*(77.99) = 15.33, *p* < .001, *d* = 3.43), and *ρ* (*t*(58.34) = 2.27, *p* = .026, *d* = 0.51, although the latter effect is considerably smaller because *ρ* is underestimated in the control group). Furthermore, this parameterization replicated the advantage of the model where resampling of rejected association is not allowed in the suppress group (ΔBIC = -133.8). Note also that this model performed worse that the no-resampling model reported in the main text (ΔBIC = 291.7)

**Parameter recovery results for the models examined in the main paper**

**
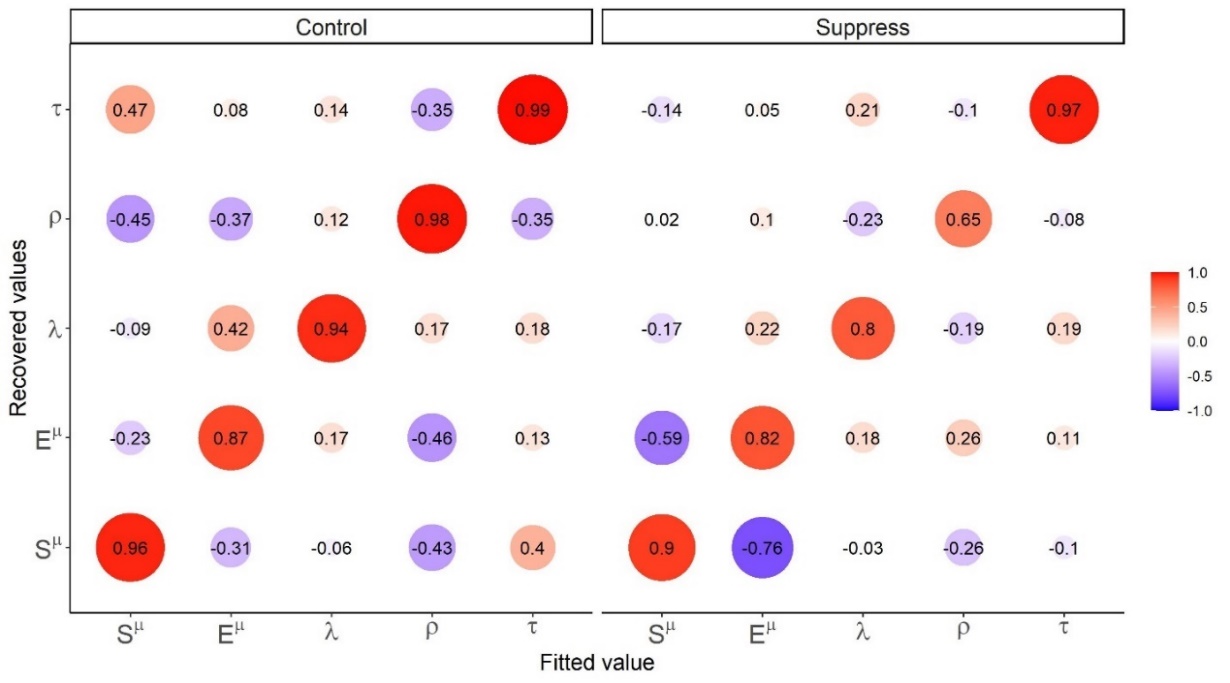
**

Fig G in S3 Text - Parameter recovery for the model with no rejections.


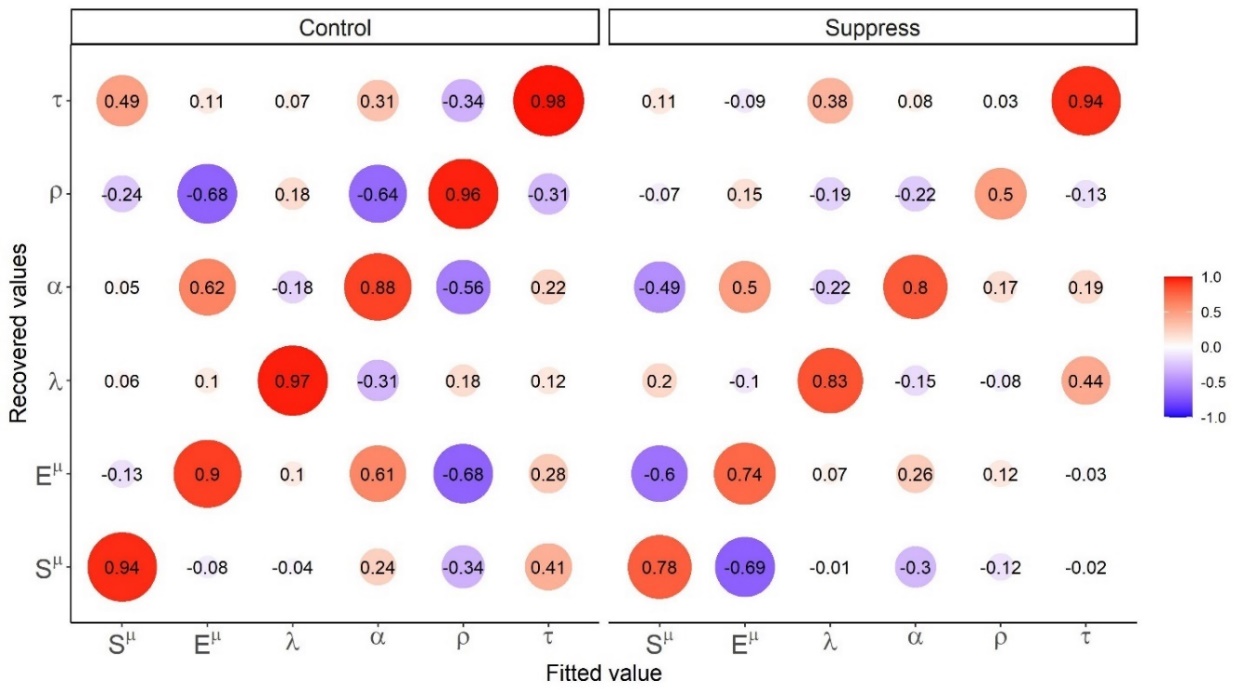


Fig H in S3 Text - Parameter recovery for a model with rejections, wherein resampling of rejected associations is allowed.

**
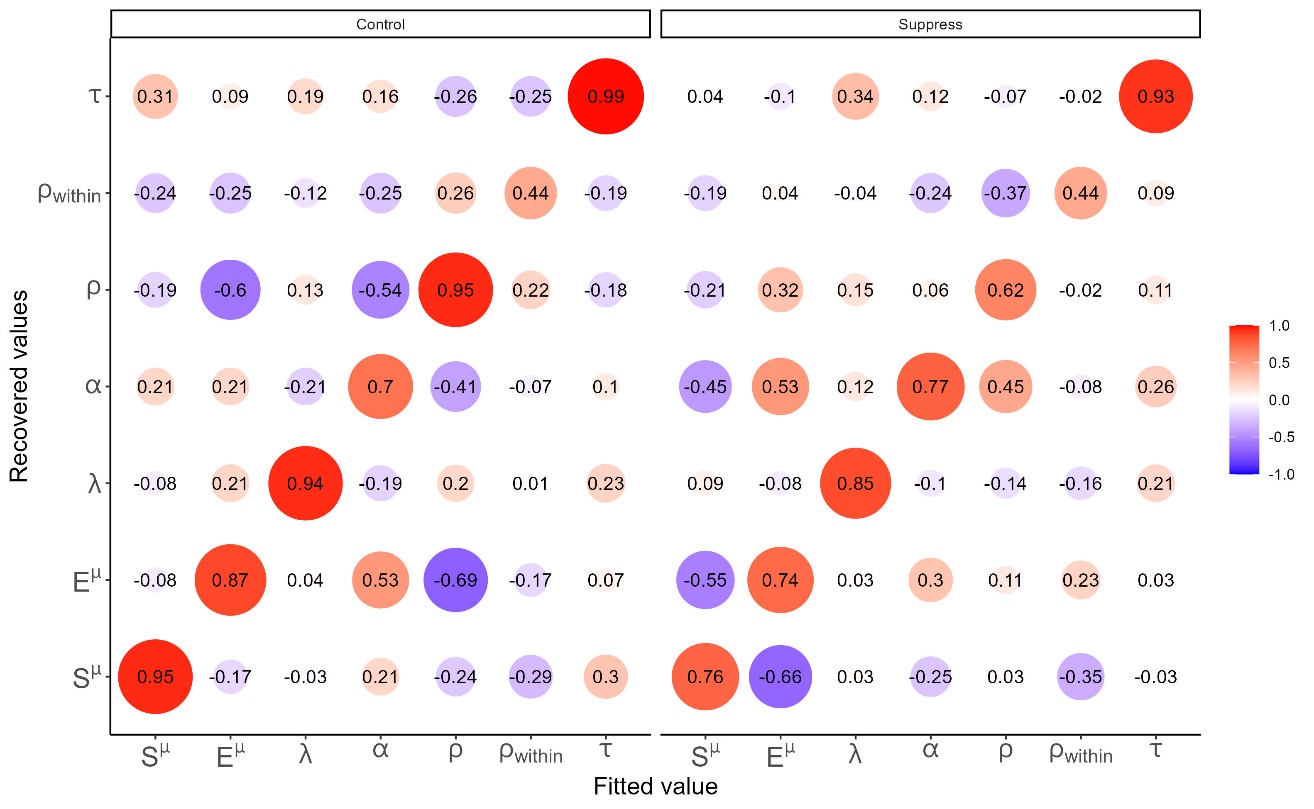
**

Fig I in S3 Text - Parameter recovery for a model with rejections, with a separate ρ_within_ (or ρ_w_) parameter modifying the associative strength of repeated associations after a first rejection).

**
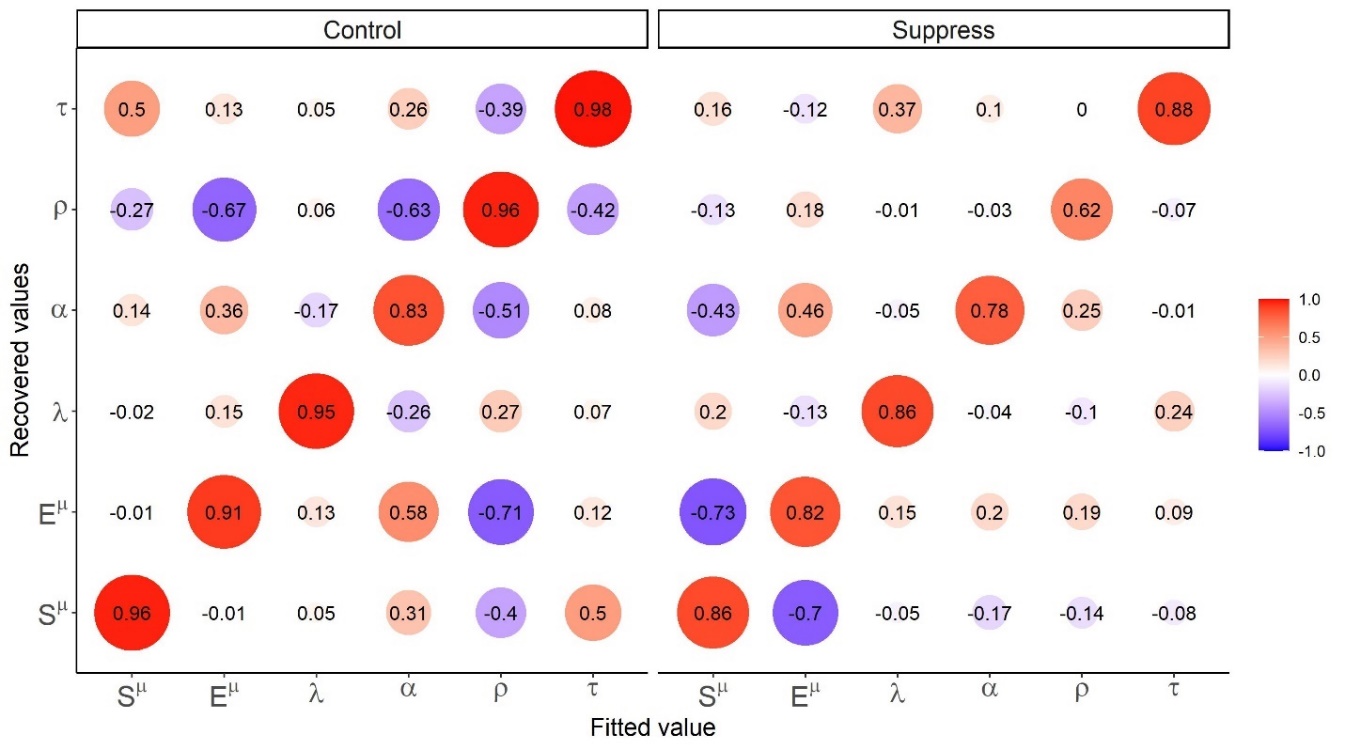
**

Fig J in S3 Text - Parameter recovery for a model with rejections, wherein resampling of rejected associations is not allowed.


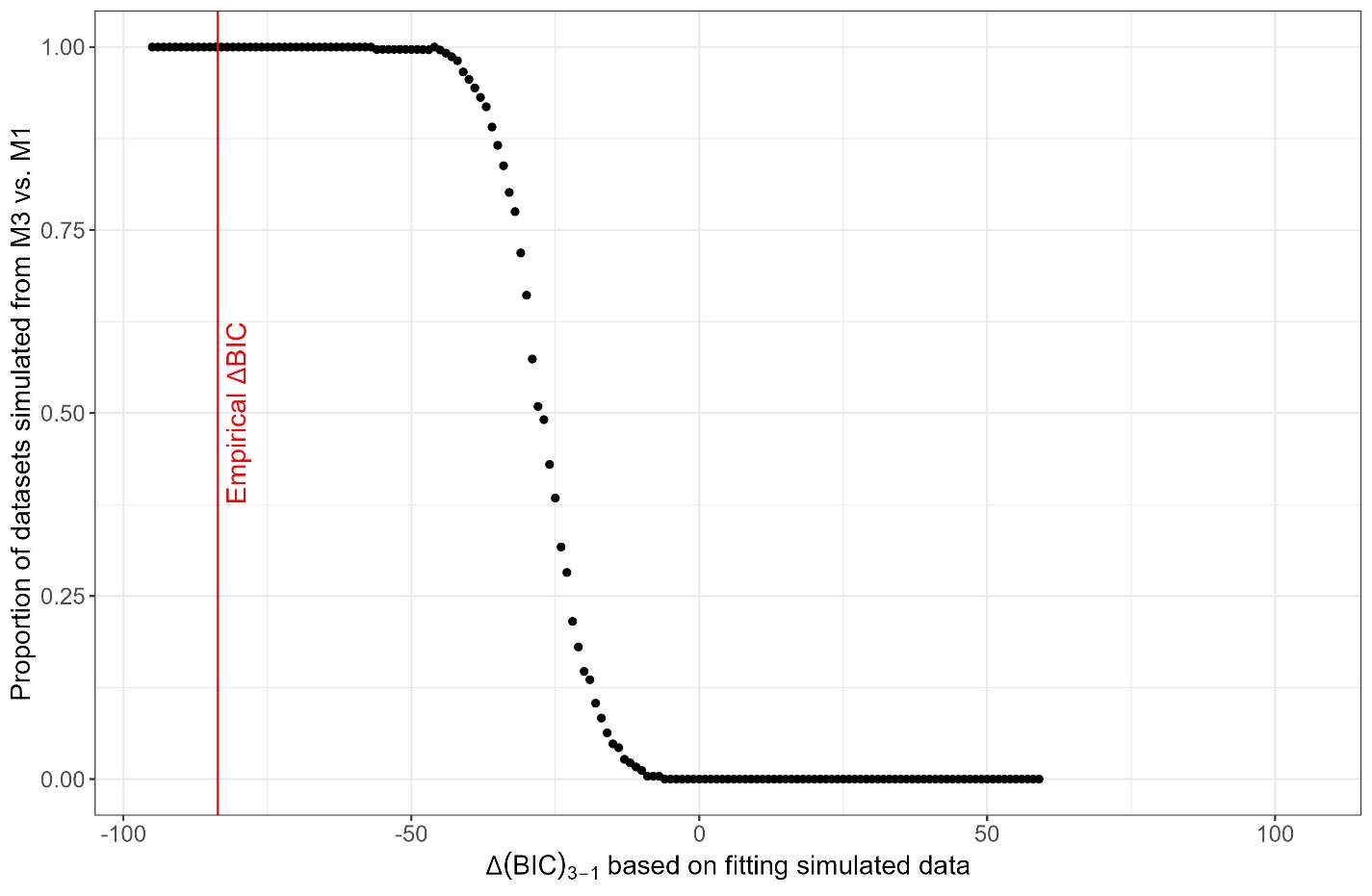


Fig K in S3 Text – The probability that the empirical ΔBIC support for a model where resampling of rejected associations is not allowed (M3) vs. a model where such resampling is allowed (M1), in the suppress group (red, vertical line), is reproduced by simulating data (1000 samples per participant) using M3 (vs. M1). Since fitting the SMP for each participant took a significant amount of time, we could not repeat the entire fitting process for each of the simulated datasets, Thus, instead, we used best-fitted parameters from either M1 and M3 to both simulate the datasets, and fit the model, varying only the question of whether resampling of rejected associations is allowed. The figure shows that a slight bias of the model comparison procedure to favor M3 over M1, even for data simulated from M1. Critically, even in cases in which data simulated from M1 results in a negative BIC (favoring M3), these values are considerably smaller than the empirical ΔBIC value.


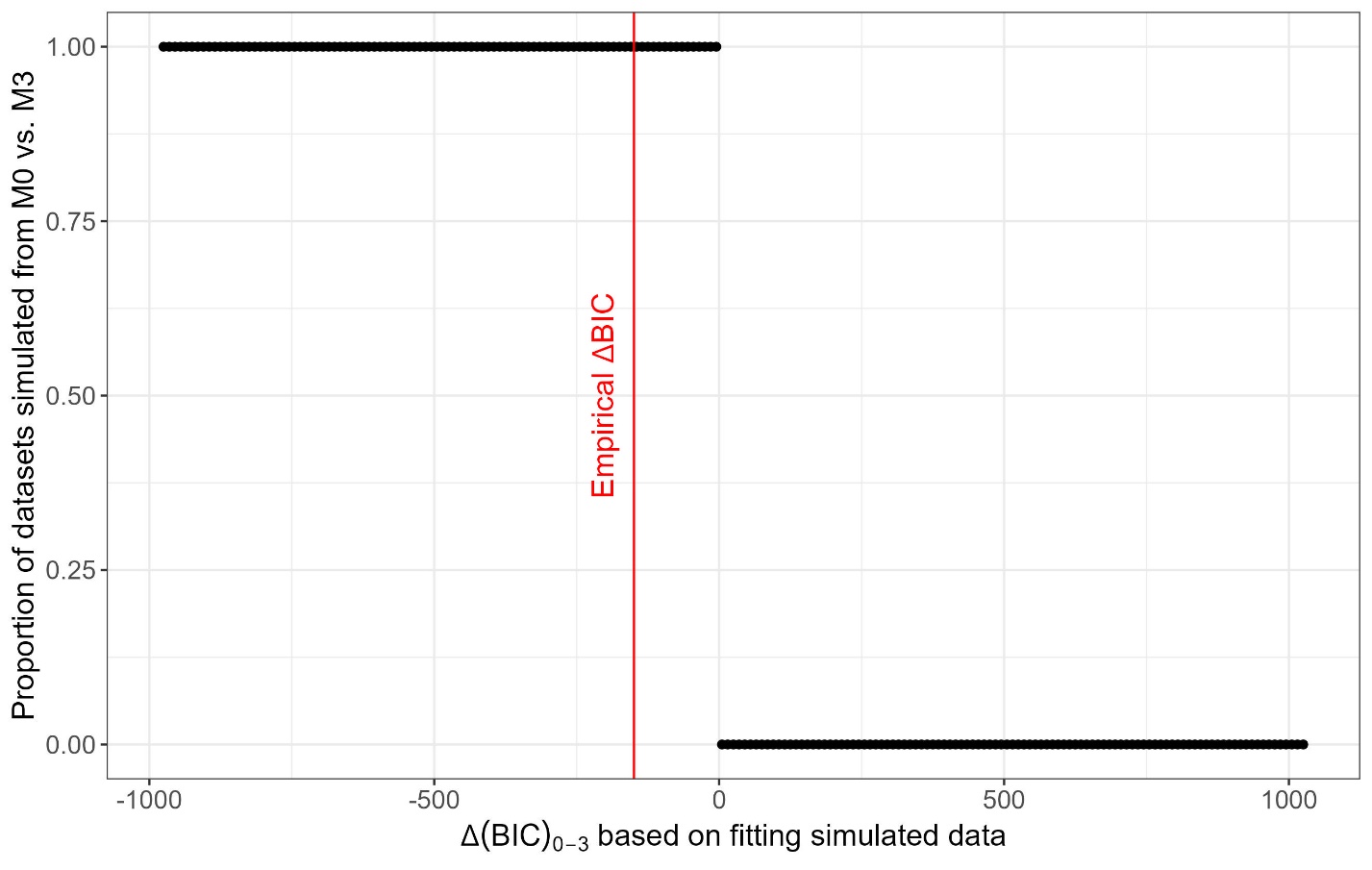


Fig L in S3 Text – The probability that the empirical ΔBIC support for a model with no rejections (M0) vs. a model with rejections, where immediate resampling of rejected associations is not allowed (M3) in the control group, is reproduced in 1000 datasets simulated using M3 (vs. M0). Because fitting the model took a significant amount of time, we could not repeat the entire fitting process for each of the simulated datasets. Thus, instead, we used best-fitted parameters from either M0 and M3 to both simulate the datasets, and fit the model, varying only the question of whether resampling of rejected associations is allowed. The results show a clear separation between the models, with no misclassifications.

**References**

[1.    Warr RL, Collins DH. An Introduction to Solving for Quantities of Interest in Finite-State Semi-Markov Processes. 2012.](https://sciwheel.com/work/bibliography/10645076)

[2.    Bergmeir C, Molina D, Benítez JM. Memetic Algorithms with Local Search Chains in *R* : TheRmalschains Package. J Stat Softw. 2016;75. doi:10.18637/jss.v075.i04](https://sciwheel.com/work/bibliography/10645173)
