# Supplementary material for: If you don’t let it in, you don’t have to get it out: Thought preemption as a method to control unwanted thoughts
Source: PLoS Comput Biol. 2022 Jul 14;18(7):e1010285. doi: 10.1371/journal.pcbi.1010285 (PMC9282588; doi:10.1371/journal.pcbi.1010285)
Supplement: S4 Text — Specification of the method used to estimate covert spaces of associations. Fig A. The distribution of normalized associative strength of different associations to the word Table, as extracted from human free association norms vs. and corpus-based cosine similarity. Fig B. The association between the data and the predictions of the model estimating the space of associations with regards to whether an association is normed or not and its rating. Fig C. Testing the sensitivity of the method we used to estimate the number and strength of non-normed associations to a different choice of bin-width. (DOCX) [file pcbi.1010285.s004.docx]

**S4 Text. Specification of the method used to estimate covert spaces of associations**

We estimated the space of possible associations by combining information from previous free association norms, with subjective associative strength ratings, and the empirical proportion of non-normed associations for each cue and participant. We briefly note here that although some studies have used corpus-based methods to derive associative strength [[1–3]](https://sciwheel.com/work/citation?ids=11079682,10645150,6292503&pre=&pre=&pre=&suf=&suf=&suf=&sa=0,0,0&dbf=0&dbf=0&dbf=0), we decided not to rely on this method for three main reasons. First, associative strength can be a highly subjective thing, with the same association having a different strength for different participants. To demonstrate this, we selected associations given by at least ten participants for a given cue and examined the range of ratings that participants provided for the same association (after a min-max normalization on ratings designed to remove individual differences in the used range of ratings). As expected, a large average range was found ([0.43, 0.99]). Second, even when compared to another population-based measure of associative strength – free association norms, corpus-based similarity distributions are considerably less lopsided (Fig A in S4 Text), suggesting that obtaining typical, high-peaked distributions from corpus-based representations, even across participants, is extremely unlikely. Third, corpus-based word similarity is usually a symmetric measure, which is inconsistent with previous findings and models of free association [[4,5]](https://sciwheel.com/work/citation?ids=3129848,4034994&pre=&pre=&suf=&suf=&sa=0,0&dbf=0&dbf=0).


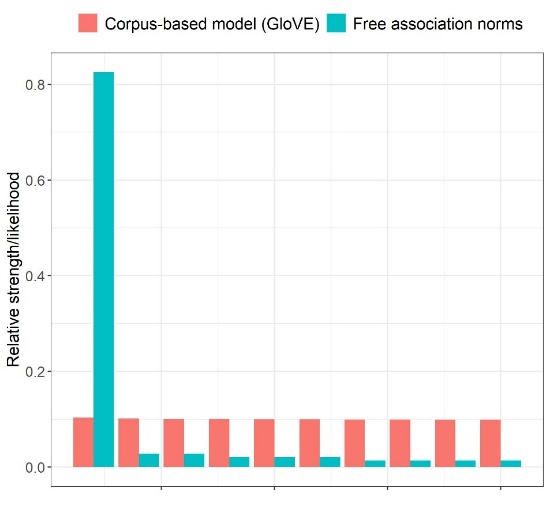


Fig A in S4 Text – The distribution of normalized associative strength of different associations to the word Table, as extracted from human free association norms vs. and corpus-based cosine similarity (limited to the 10 strongest responses). Corpus-based similarity was calculated using pre-trained GloVe word embeddings, with 300 dimensions. The substantial difference between how human-based associations are distributed and how corpus-based similarity is distributed demonstrates one reason for not using corpus-based measures to derive the spaces of possible associations in our task.

We now describe the method we used to estimate the number and strength of personalized association. First, we examine the problem of estimating the number of distinct associations for a given cue not appearing in previous norms (non-normed associations). For a given cue, let n(*K*) and n(*U*) denote the number of normed and non-normed possible associations, and let *p*(*k*) and p(*u*) represent the probabilities of *reporting* a normed and a non-normed association, respectively (where *p(u)*+*p(k)* = 1*)*. Since non-normed associations tend to generally have lower associative strength (in a model predicting the probability for a non-normed association, given associative strength rating: *β* = -3.92, 95% Bayesian CI [-4.19, -3.64], odds ratio = 0.01), we condition the probability of reporting normed and non-normed associations on associative strength (AS). Conditional on associative strength, we can determine the number of distinct non-normed associations based on the number of distinct normed associations, and the likelihood of reporting a non-normed association:

$$\begin{aligned} n\left( U,AS \right)=n\left( K,AS \right)\frac{p\left( u|AS \right)}{p\left( k|AS \right)}\#A \end{aligned}$$

Because associative strength is continuous, we model the odds of reporting a non-normed association as a function of associative strength rating using mixed-effects logistic regression. Using this approach obviates the need to collect multiple associations with equal associative strength for each cue and participant, and yet, can account for differences between participants (*s*) and between cues (*c*) in the odds of reporting a non-normed association:

$$\begin{aligned} \log\left( \frac{p\left( u|AS,s,c \right)}{p\left( k|AS,s,c \right)} \right)=\beta_{0}+\beta_{0|s}+\beta_{0|c}+\left( \beta_{1}+\beta_{1|s}+\beta_{1|c} \right)\cdot{AS}_{s,c}.\#B \end{aligned}$$

Once the odds of reporting a non-normed association are inferred, they can be used to estimate the corresponding number of non-normed associations for each participant and cue (see Equation A in S4 Text). This also requires us to know associative strength for both reported associations (for which we have participants' ratings) and unreported normed associations. To estimate the latter, we rely on the relationship between associative strength ratings and the proportion of participants reporting each normed association in previous norms (pTP). Thus, we model the expected AS of association $a\in\{1,2..n(k)\}$ of participant *s* in response to cue *c* as:

$$\begin{aligned} \mu_{{AS}_{a,s,c}}=\beta_{0}+\beta_{0|s}+\beta_{0|c}+\left( \beta_{1}+\beta_{1|s}+\beta_{1|c} \right)\cdot pTP_{a,c}.\#C \end{aligned}$$

To account for the limited scale of AS ratings [0,1], we assume it is sampled from a rectified normal distribution. We examined the extent to which these models (Equations B-C in S4 Text) correctly predict participants' tendency to report non-normed associations (p(u)) and the ratings of normed, reported associations (AS ratings). As shown in Figure B in S4 Text (Panel A), the model correctly predicted whether a reported association is non-normed for most participants. The median Pearson correlation (within participants) between empirical ratings and model-predicted ratings for normed associations was 0.34 (Q_25_ = 0.28, Q_75_ =0.43). Figure B in S4 Text (Panel B) depicts this relationship (both variables are person-scaled to focus on this correlation within participants and partial out individual differences in averages). Together, these results support the ability of the estimation method delineated above to provide a satisfactory account of the key response variables (AS and p(u)).


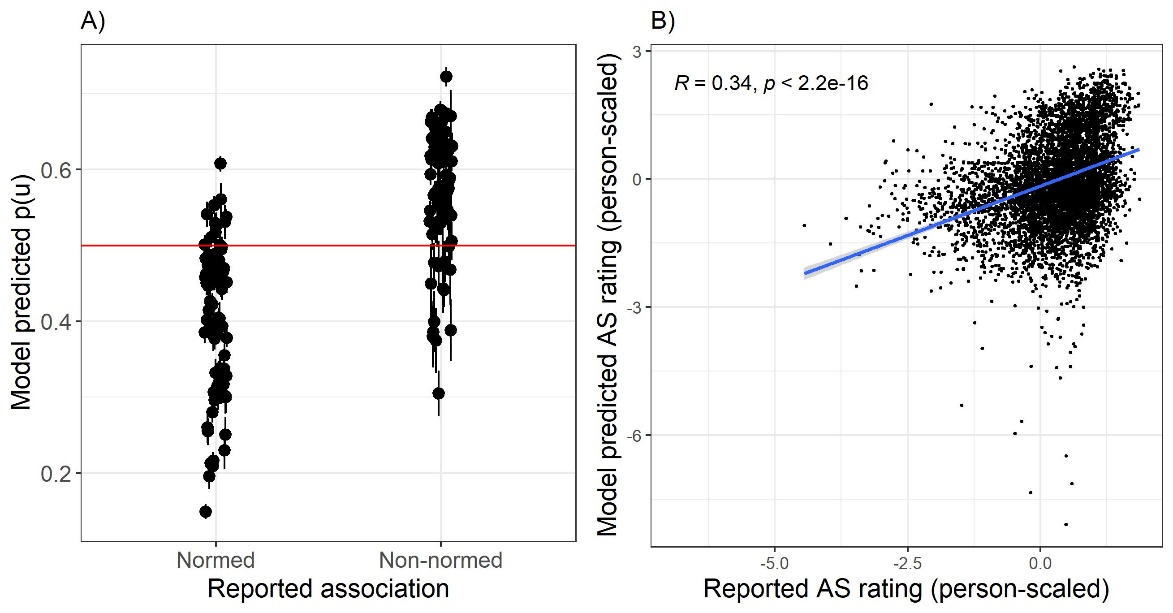


*Fig B in S4 Text – the association between the data and the predictions of the model estimating the space of associations with regards to whether an association is normed or not (panel A; each point represents one participant), and its rating (panel B; for normed associations only).*

Thus, the above procedure allows us to estimate the associative strength of each possible normed association (Equation C in S4 Text) and the odds that an association is non-normed for each possible AS (Equation B in S4 Text). Using these, we can approximate the number and strength of non-normed associations. To do this, we split the range of AS into small bins (of size 0.02; using smaller bins did not alter the results; see Figure C in S4 Text) and then estimate via sampling the number of normed associations within each bin, denoted by $\theta_{bin,s,c}^{(k)}$. Then, the estimated number of non-normed associations within each bin is given by:

$$\begin{aligned} \theta_{bin,s,c}^{\left( u \right)}=\theta_{bin,s,c}^{\left( k \right)}\frac{p\left( u|AS_{bin},s,c \right)}{1-p\left( u|AS_{bin},s,c \right)},\#D \end{aligned}$$

where $AS_{bin}$ is the midpoint of the range covered by the bin.


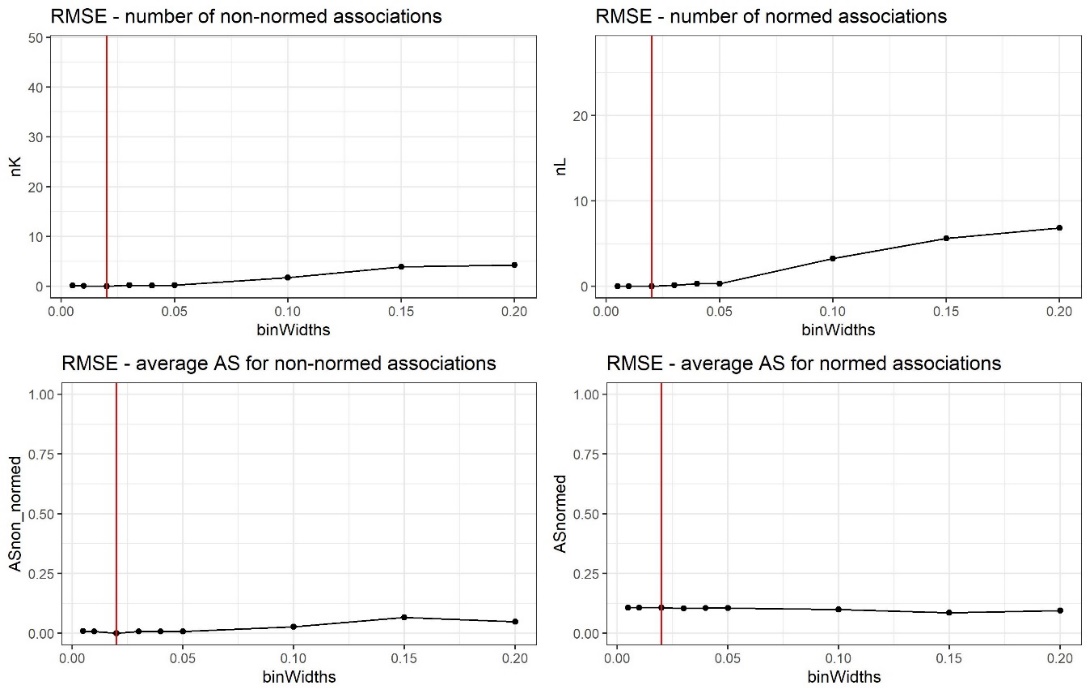


Fig C in S4 Text – Testing the sensitivity of the method we used to estimate the number and strength of non-normed associations to a different choice of bin-width, used to separate continuous predictions of the models to a discrete number of associations. Whereas a bin-width of 0.02 was used in the main text, this sensitivity analysis has shown that choosing other, small enough (< 0.05) values for the bin-width produced largely similar association spaces. The figure presents the root mean squared error (RMSE) resulted from using other bin-widths (compared with the bin-width used in the main text – 0.02, marked by red, horizontal line)

Finally, to allow for the modeling of a decision process that selects among the inferred space of associations, it is helpful to discretize the non-integer numbers of expected associations, $\theta_{bin,s,c}$, into integer counts. For this purpose, we rounded $\theta_{bin,s,c}$ downwards and set each association's strength to $AS_{bin}$. To account for the remainders, we derived an additional association wherever the sum of remainders over consecutive bins crossed 1. The AS for these associations was computed as a weighted average of the traversed $AS_{bin}$, with the remainders serving as weights

Both mixed-effects models (Equations B-C in S4 Text) were estimated jointly in a hierarchical Bayesian model, using Hamiltonian MCMC as implemented in Stan, with non-central parameterization [[6]](https://sciwheel.com/work/citation?ids=3442496&pre=&suf=&sa=0&dbf=0), and weakly information priors. The MCMC included three chains, with 10,000 iterations each, 1,000 of which were discarded during warm-up to calibrate the Hamiltonian parameters. No divergent transitions were detected, indicating unbiased sampling. Similarly, all models converged as indicated by $\hat{R}$ values the did not exceed 1.01.

Next, we focus on demonstrating the validity of our method. First, we examined the reliability of the estimation method above by naively sampling reported associations (for each participant and cue) from the estimated association space (in proportion to the estimated AS distribution) and using the same estimation method on this simulated data to obtain recovered association spaces (for each cue and participant). This analysis has shown a very strong correlation between the estimated and the recovered number of non-normed associations ($n_{s,c}\left( U \right);r=0.91)$, as well as strong correlations between the estimated and recovered values of the average AS (averaged within trials) for non-normed associations (*r* = 0.83). Finally, examining the difference between estimated and recovered association spaces at the level of specific participants and cues, using the Kolmogorov-Smirnov test has shown evidence for a statistically significant difference in only 5.82% of the trials, which is only slightly above chance level (i.e., the expected false positive rate of 5%)

Next, to verify the importance of considering the role of non-normed associations in the association space, we examined the hypothesis that a larger number of possible non-normed association will predict slower RTs (due to increased competition), even when controlling for the number of normed associations, and the identity of the reported association (i.e., normed or non-normed). Indeed, a mixed-effects model has shown a significant effect for the (standardized) number of possible normed associations in predicting log RT (β = 0.13, SE = 0.02, t (70.21) = 7.20, p < .001). This effect was found whether the reported association was normed or not. Furthermore, in trials in which a non-normed association was reported, this effect was stronger (β = 0.09, SE = 0.03, t (58.66) = 3.42, p = .001) than the effect of the number of normed associations (β = 0.05, SE = 0.02, t (133.81) = 2.19, p = .03). These findings clearly show that association generation dynamics are related to the number of potential non-normed associations (such that ignoring those will worsen the investigation of these retrieval dynamics) and that our estimation procedure successfully provides a valid estimate for this number.

**References**

[1.    Gray K, Anderson S, Chen EE, Kelly JM, Christian MS, Patrick J, et al. “Forward flow”: A new measure to quantify free thought and predict creativity. Am Psychol. 2019;74: 539–554. doi:10.1037/amp0000391](https://sciwheel.com/work/bibliography/11079682)

[2.    Avery J, Jones MN. Comparing models of semantic fluency: Do humans forage optimally, or walk randomly? Proceedings of the 41st Annual Conference of the Cognitive Science Society. 2019; 118–123.](https://sciwheel.com/work/bibliography/10645150)

[3.    Hills TT, Jones MN, Todd PM. Optimal foraging in semantic memory. Psychol Rev. 2012;119: 431–440. doi:10.1037/a0027373](https://sciwheel.com/work/bibliography/6292503)

[4.    Griffiths TL, Steyvers M, Tenenbaum JB. Topics in semantic representation. Psychol Rev. 2007;114: 211–244. doi:10.1037/0033-295X.114.2.211](https://sciwheel.com/work/bibliography/3129848)

[5.    Nelson DL, Dyrdal GM, Goodmon LB. What is preexisting strength? Predicting free association probabilities, similarity ratings, and cued recall probabilities. Psychon Bull Rev. 2005;12: 711–719. doi:10.3758/bf03196762](https://sciwheel.com/work/bibliography/4034994)

[6.    Carpenter B, Gelman A, Hoffman MD, Lee D, Goodrich B, Betancourt M, et al. *stan* : A probabilistic programming language. J Stat Softw. 2017;76: 1–32. doi:10.18637/jss.v076.i01](https://sciwheel.com/work/bibliography/3442496)
