# Supplementary material for: If you don’t let it in, you don’t have to get it out: Thought preemption as a method to control unwanted thoughts
Source: PLoS Comput Biol. 2022 Jul 14;18(7):e1010285. doi: 10.1371/journal.pcbi.1010285 (PMC9282588; doi:10.1371/journal.pcbi.1010285)
Supplement: S5 Text — Specification of an adapted SMP model with either proactive or reactive inhibition of weak (i.e., uncommon) associations. Table A.–Supplementary model comparison results. (DOCX) [file pcbi.1010285.s005.docx]

**S5 Text. Specification of an adapted SMP model with either proactive or reactive inhibition of weak (i.e., uncommon) associations**

This extension of the SMP was designed to account for potential control mechanisms that participants might have used to comply with the instruction to provide only strong and common associations. Proactive control was formalized by assuming that the probability of selecting strong association could have been further increased, by means of modifying the latent associative strength (*lAS*) of stronger associations:

$$\begin{aligned} lAS_{i}=\frac{AS_{i}^{e^{\eta}}}{\sum_{i\in I} AS_{i}^{e^{\eta}}}*\sum_{i\in I} AS_{i}\#A \end{aligned}$$

where I is the number of associations for a cue, and the multiplication by $\sum_{i\in I} AS_{i}$ is designed to verify that this transformation changes the relative strength of the different associations but not the overall absolute strength. Thus, when $\eta$ is positive, strong associations become even stronger, and weak associations become weaker. The result of Equation A in S5 Text then replaces AS_i_ in Equation 1 in the main text.

Reactive control over weak associations was formulized by setting the probability of *accepting* any association to be a function of both its associative strength (after a min-max transformation, designed to verify that acceptance is governed by the relative strength of an association, but is not affected by the number of associations. We denote this transformation as MX). Thus, the probability of accepting a non-repeated association was given by:

$$\begin{aligned} p\left( accepting | A_{i} \right)=\frac{1}{1+e^{-10\left[ MX\left( AS_{i} \right)-\alpha_{AS} \right]}}\#B \end{aligned}$$

Where $\alpha_{AS}$ is a free parameter determining the probability of rejecting weak associations. Thus, the probability of accepting a repeated association was simply given by multiplying the result of Equation B in S5 Text by 1-α, where α is a free parameter determining the probability of rejecting repeated associations.

Together this produces three possible models (with only proactive AS control, only reactive AS control or both), that were combined with the winning models reported in the main text for each group (a model with no repetition-based rejections for the control group, and a model with repetition-based rejections and no immediate resampling of rejected associations for the suppress group). The results of comparing these models to the models with no AS control that were found to perform best for each group (see Table 1 in the main text) are reported in Table A in S5 Text. The results suggest that participants enacted AS control, but also suggest that this type of control was primarily proactive.

| **Table A in S5 Text – Supplementary model comparison results.** The table shows Bayesian Information Criteria Difference in fitting the experimental data from the two study groups, while considering proactive and reactive control over weak associations (*AS control*), relative to the model that best fitted the data reported in the main text. | | |
| --- | --- | --- |
| **Model** | **Suppress group** | **Control group** |
| Proactive AS-control | -1135.04 | -1431.41 |
| Reactive AS-control | -969.27 | -903.65 |
| Proactive and reactive AS-control | -1067.95 | -1414.43 |
