# Supplementary material for: If you don’t let it in, you don’t have to get it out: Thought preemption as a method to control unwanted thoughts
Source: PLoS Comput Biol. 2022 Jul 14;18(7):e1010285. doi: 10.1371/journal.pcbi.1010285 (PMC9282588; doi:10.1371/journal.pcbi.1010285)
Supplement: S1 Fig — (DOCX) [file pcbi.1010285.s006.docx]

**Figure S1.**


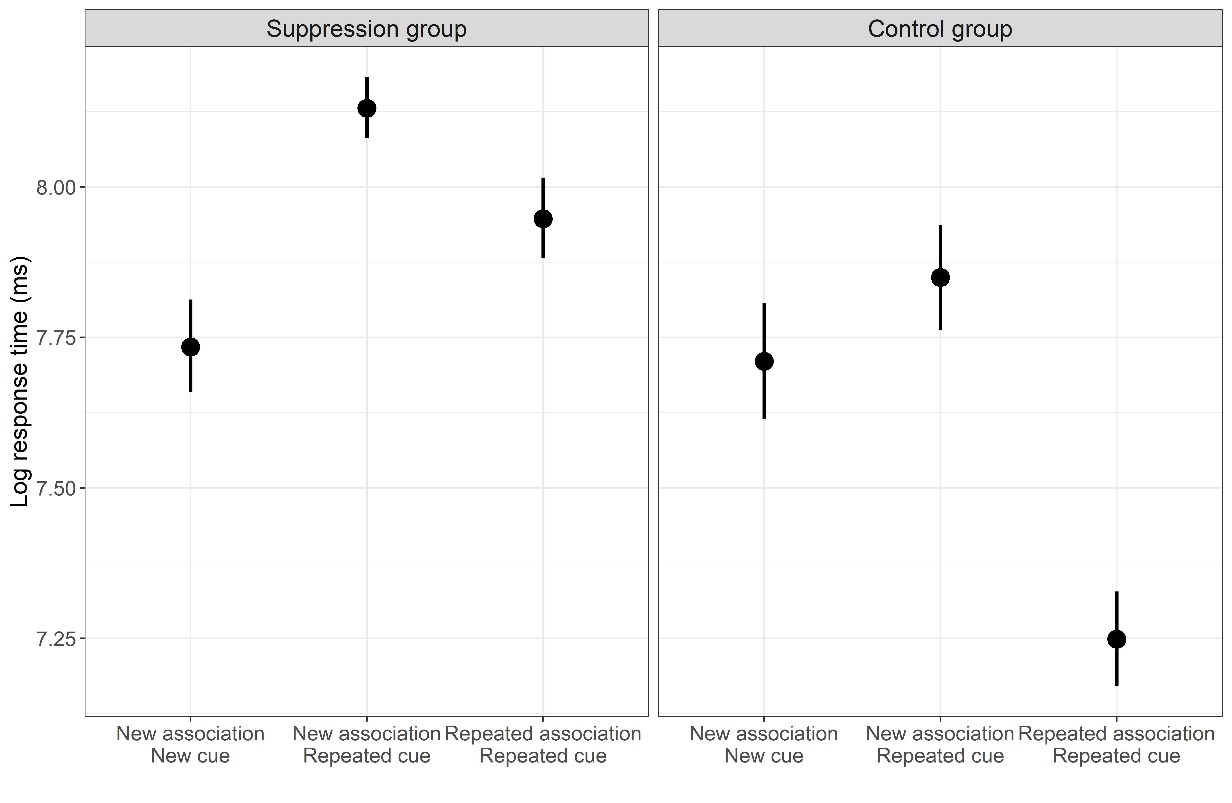


Figure S1 – Sensitivity analysis – Testing the effect of excluding participants with exceptionally low variance in ratings. The figure presents mean (± 95% bootstrapped confidence interval) log response times for the two groups, and different trial types. Replicating the result reported in the main test, and as predicted by reactive thought control, the attempt to avoid repeated associations resulted in a larger increase in average RTs (t (80.40) = 4.67, p < .001, Cohen’s d = 1.02).
