# Supplementary material for: If you don’t let it in, you don’t have to get it out: Thought preemption as a method to control unwanted thoughts
Source: PLoS Comput Biol. 2022 Jul 14;18(7):e1010285. doi: 10.1371/journal.pcbi.1010285 (PMC9282588; doi:10.1371/journal.pcbi.1010285)
Supplement: S2 Fig — (DOCX) [file pcbi.1010285.s007.docx]

**S2 Figure. Best-fitted parameter values in the two groups**

**
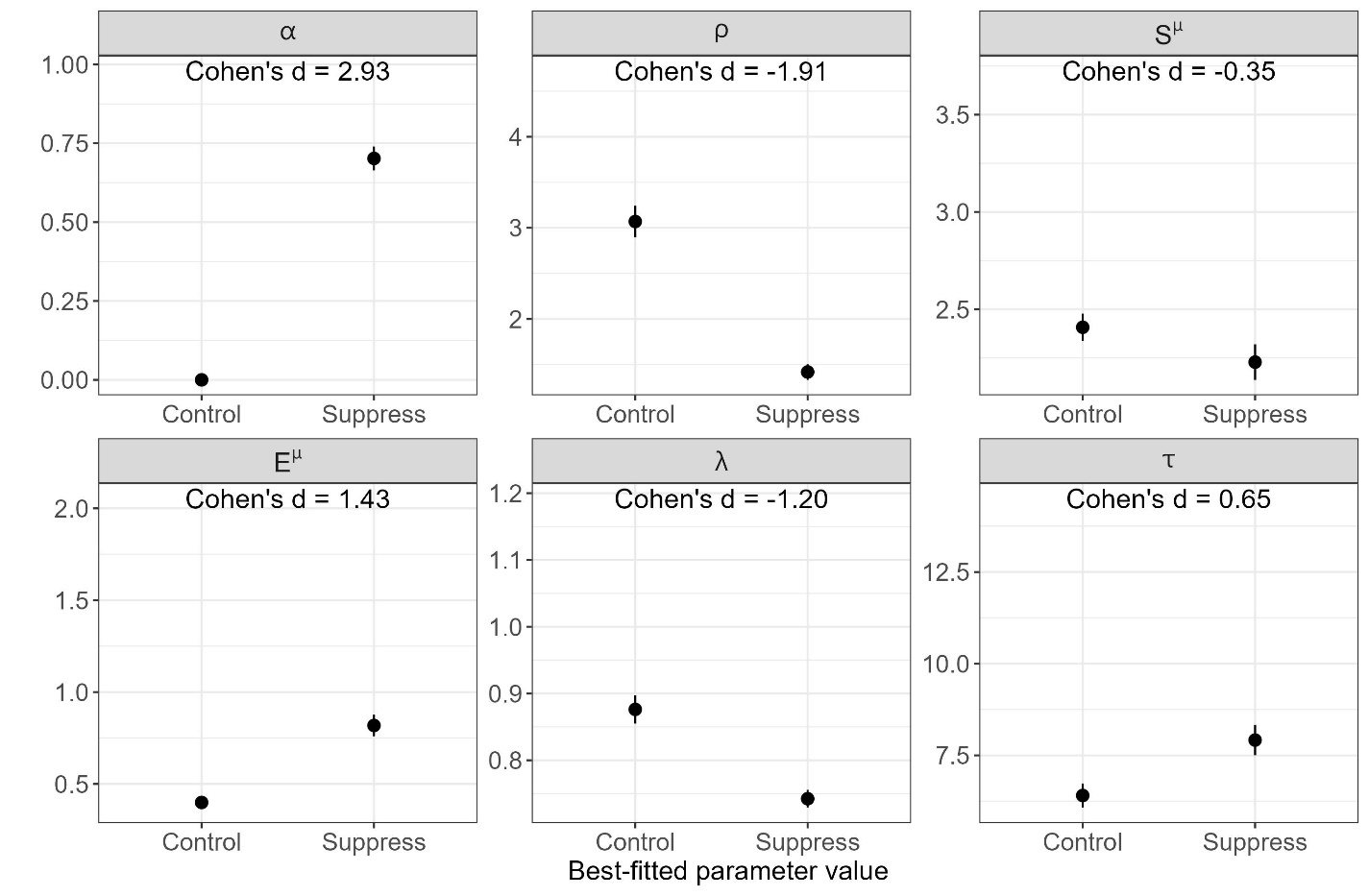
**

Figure S2 – Best-fitted parameter values in the two groups (based on winning models in both groups; see Table 1 in the main text).
