# Supplementary material for: If you don’t let it in, you don’t have to get it out: Thought preemption as a method to control unwanted thoughts
Source: PLoS Comput Biol. 2022 Jul 14;18(7):e1010285. doi: 10.1371/journal.pcbi.1010285 (PMC9282588; doi:10.1371/journal.pcbi.1010285)
Supplement: S3 Fig — (DOCX) [file pcbi.1010285.s008.docx]

**S3 Figure.**

**
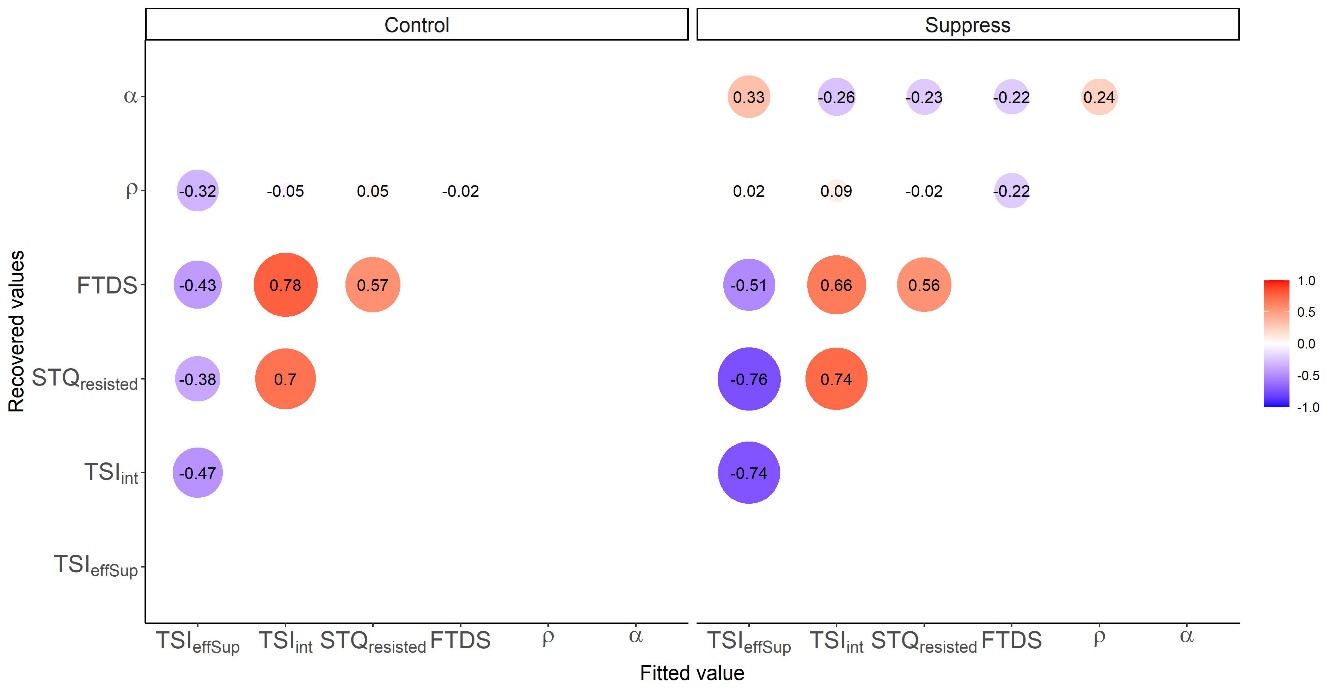
**

S3 Figure – Correlation matrix of the different self-report measures and the SMP model parameters, examined as an exploratory analysis. Following our preregistration, in the paper we focus on TSI-R effective suppression subscale (TSI-effSup). However, given the exploratory nature of this analysis, we also wanted to examine whether the mere experience of having unwanted thoughts correlates with model parameters. This was measured by both the TSI intrusions subscale, and the resisted-thoughts subscale of the Surprising Thoughts Questionnaire[[1]](https://sciwheel.com/work/citation?ids=10644882&pre=&suf=&sa=0&dbf=0), both measuring self-reported experiences of having unwanted thoughts. We also explored the correlations with the Formal Thought Disorder scale – Revised[[2]](https://sciwheel.com/work/citation?ids=3962122&pre=&suf=&sa=0&dbf=0), measuring disordered thinking. Given that these scales are negatively correlated with the TSI-R effective suppression subscale, it is not surprising to find correlations between α and these additional questionnaires, all suggesting that people experiencing their thoughts as less controllable also have a greater difficulty inhibiting repeated associations in the task. Conversely, the relationship between ρ and TSI-effSup appears to be much more specific.

**References**

[1.    Fradkin I, Huppert JD. When our train of thought goes off track: The different facets of out-of-context thoughts in obsessive compulsive disorder. J Obsessive Compuls Relat Disord. 2018;18: 31–39. doi:10.1016/j.jocrd.2018.06.001](https://sciwheel.com/work/bibliography/10644882)

[2.    Barrera A, McKenna PJ, Berrios GE. Two new scales of formal thought disorder in schizophrenia. Psychiatry Res. 2008;157: 225–234. doi:10.1016/j.psychres.2006.09.017](https://sciwheel.com/work/bibliography/3962122)
